# Cause of death diversity in multi-group settings: an application to Latin America and the Caribbean

**DOI:** 10.1186/s12963-025-00436-3

**Published:** 2025-12-08

**Authors:** Júlia Almeida Calazans, Iñaki Permanyer

**Affiliations:** 1 Center for Demographic Studies (CED-CERCA), Universitat Autònoma de Barcelona, Bellaterra, Spain; 2 Institució Catalana de Recerca i Estudis Avançats (ICREA), Universitat Autònoma de Barcelona - Bellaterra, Barcelona, Spain

**Keywords:** Causes of death, Mortality, Diversity indicators, Decomposition methods

## Abstract

**Background:**

Cause of death (CoD) diversity indices measure the extent to which some populations die from more similar or variegated causes than others. Higher CoD diversity implies higher unpredictability of the causes of individuals dying and greater challenges for health systems. In this paper, we propose a novel method to decompose overall CoD diversity as the sum of two interpretable parts: the within- and between-group components.

**Methods:**

The novel approach is applied to Latin America and the Caribbean (LAC) region to illustrate its usefulness. We decompose overall CoD diversity, measured by the Simpson index of diversity, into between-country and within-country components. In addition, we provide further decompositions assessing how each cause of death and each country contributes to overall CoD diversity in the region.

**Results:**

The CoD diversity in the region followed a nonmonotonic trend. From 2000 to 2018, the CoD diversity increased from 0.81 to 0.83 for women, reaching approximately 0.84 for men. El Salvador, Peru, and Uruguay are the countries that contribute the most to explaining the differences in the mortality profile between countries, but for very different and opposing reasons. While the high diversity in El Salvador and Peru can be explained by causes of deaths related to the early stages of the epidemiological transition, such as communicable causes, respiratory causes, and external causes, Uruguay presents a high diversity because the deaths are very dispersed between chronic conditions. Cardiovascular deaths are the main contributor to both CoD diversity levels and their changes over time. As cardiovascular deaths decline, they give way to other chronic causes, which become more prominent and contribute to diversifying the corresponding mortality profiles. However, external causes also significantly contribute to forming uneven epidemiological profiles.

**Conclusions:**

The decomposition proposed in this paper makes possible to assess whether some groups contribute more or less to the uncertainty around the causes of individuals’ deaths and identify the sources of CoD diversity. In this way, this approach can contribute to a better understanding of contemporary mortality dynamics, especially in a context with large health inequalities.

**Supplementary Information:**

The online version contains supplementary material available at 10.1186/s12963-025-00436-3.

## Background

Cause of death (CoD) diversity indices measure the extent to which some populations die from more similar or variegated causes than others. The greater the diversity in CoD is, the higher the unpredictability of the causes from which individuals will die. Keeping other factors constant, higher CoD diversity implies greater challenges for health systems, making efforts to reduce total mortality more complex and possibly less effective [1, 2]. For these reasons, CoD diversity indicators have recently been recognized as important measures of population health and well-being, providing new perspectives on our understanding of contemporary mortality dynamics [1–3].

Importantly, current approaches to measure CoD diversity have never attempted to assess whether, in the presence of population groups (e.g., along geographical, socioeconomic, racial, ethnic, gender or other lines), the diversity in CoD observed for the entire population is mostly attributable to the CoD diversity that might exist *within* the groups conforming to that group or the diversity in CoD that might exist *between* them. This is an important limitation, hindering the possibility of having an overall reliable picture of the extent of diversity in mortality profiles in the presence of well-defined population subgroups with specific epidemiological profiles. In this paper, we propose a novel method to decompose overall CoD diversity as the sum of two clearly interpretable parts: the within- and between-group components. The first one is a weighted sum of the CoD diversity levels within the groups conforming to the population partition, while the second one captures the diversity one can observe when comparing the mortality profiles among the different groups. In addition, we provide further decompositions assessing how each cause of death and each group contributes to overall CoD diversity. These decompositions allow the determination of the main sources of CoD diversity, thus providing a useful tool for analysists and policy-makers aiming to identify and eliminate health inequalities.

To illustrate the usefulness of our novel approach, we will apply it to the context of Latin America and the Caribbean (LAC), one of the regions with the highest health inequalities worldwide. Health inequalities refer to disparities that are unnecessary, unjust, and avoidable [4]. They stem not only from unequal access to healthcare services and differences in living conditions but also from complex social dynamics [4, 5]. These inequities—whether demographic, social, economic, or geographic—ultimately contribute to significant disparities in life expectancy and cause-specific mortality patterns.

The causes of death in Latin America and the Caribbean have been studied extensively over the last few decades, as have the regional similarities and differences in the epidemiological transition process [6–17]. Notably, it is well known that several countries in the region have been unable to complete certain phases of the epidemiological transition [18]. As documented elsewhere, there is an overlap of a high incidence of diseases from the pretransitional stage – infectious diseases and external causes – and posttransitional stage – chronic diseases – without a clear resolution of each stage of the transition process. For this reason, Frenk et al. [8] state that the epidemiological transition process in Latin America and the Caribbean follows a “polarized-prolonged model”.

This coexistence can be explained, to a large extent, by the persistent socioeconomic differences that exist in the countries of the region. Prata [19] states that wealthier population groups have greater access to health services, better housing conditions, and better nutritional conditions and are therefore in the more advanced stages of the epidemiological transition, while the most vulnerable groups are in the early stages, with higher mortality rates from infectious diseases and external causes. The overlap in stages of the epidemiological transition leads to very heterogeneous mortality profiles. In a recent study, Calazans and Permanyer [2] identified Latin America and the Caribbean as regions with higher CoD diversity around the globe. Furthermore, the region presents an increase in CoD diversity over time because diseases such as neoplasms or neurological disorders are becoming increasingly prevalent.

Comparative studies on the heterogeneity in mortality profiles among and within the countries of the region are lacking. This paper analyses the extent of diversity in the mortality profile in a group of 21 countries in Latin America and the Caribbean. First, we decompose overall CoD diversity into between-country and within-country components, and then we analyze the contribution of each country and each cause of death to overall CoD diversity and to each of these two components.

## Methods

### Data

CoD diversity measures are based on the share of deaths attributable to the different causes one is working with. The probability of dying from each cause was calculated using the age-and-cause-specific number of deaths from the Mortality Database organized by the World Health Organization [20] and the age-specific mortality rates from the World Population Prospects [21]. Deaths were classified into 18 major groups of underlying causes of death following the chapters of the 10th Revision of the International Classification of Diseases (ICD-10). There were four communicable causes: infectious diseases (A00-B99), pregnancy, childbirth, and puerperium (O00-O99), perinatal conditions (P00-P96) and congenital malformations, deformations, and chromosomal abnormalities (Q00-Q99). There were twelve noncommunicable causes of death: neoplasms (C00-D48), cardiovascular diseases (I00-I99), blood diseases (D50-D89), endocrine diseases (E00-E90), mental disorders (F00-F99), neurological diseases (G00-G99), diseases of the eye and ear (H00-H59 and H60-H95), respiratory diseases (J00-J99), digestive diseases (K00-K93), skin diseases (L00-L99), musculoskeletal diseases (M00-M99), genitourinary diseases (N00-N99), one external causes group (S00-T98 and V01-Y98) and one ill-defined causes group (R00-R99).

The analysis was carried out for a group of 21 countries in Latin America and the Caribbean distributed in the Caribbean (Antigua and Barbuda, Cuba, Dominica, Dominican Republic, Grenada, Saint Lucia, and Saint Vincent and Grenadines), Central America (Costa Rica, El Salvador, Mexico, Nicaragua and Panama) and South America (Argentina, Brazil, Chile, Colombia, Ecuador, Guyana, Paraguay, Peru and Uruguay). Countries with long historical data series were selected, but we also sought to ensure that the selected set included countries with different socioeconomic conditions and mortality levels. Furthermore, these countries account for 85% of the region’s total deaths. Data on the number of deaths by causes of death were not available for Antigua and Barbuda (2010, 2011), Cuba (2000), Dominica (2000), the Dominican Republic (2019), Grenada (2000), Saint Lucia (2007) and Uruguay (2011). They were imputed as a linear interpolation of the values observed by sex, age group, and cause of death.

### Measuring CoD diversity

In this paper, CoD diversity is measured using the Simpson index of diversity, $\:S$. Assuming that all deaths are classified into a list of $\:k$ mutually exclusive causes, $\:S$ is defined as the probability that two randomly chosen deaths are attributable to different causes. Formally, it is defined as

1 $$\:S=1-\:\sum\:_{c=1}^{k}{p}_{c}^{2}$$

where $\:{p}_{c}$ is the share of deaths attributable to cause $\:c$ in the total deaths. The CoD diversity index is calculated based on the causes of death distribution from the corresponding life tables (rather than the observed number of deaths) to render populations with different age structures comparable.

Lower values of $\:S$ indicate that deaths are increasingly concentrated due to fewer causes. In the limit, if all individuals died from the exact cause, $\:S$ would equal zero. At the other extreme, higher values of $\:S$ indicate that the causes from which individuals die become increasingly diverse. The $\:S$ index is maximized when deaths are equally distributed across all possible causes (when this happens, $\:S$ equals $\:(k-1)/k$, which corresponds to $\:17/18\cong\:0.94$ in our setting).

### CoD diversity in multigroup settings

We assume that our population is divided across $\:G$ mutually exclusive subgroups, in our case, countries. An attractive feature of Simpson’s diversity index $\:S$ is that it can be broken down as

2 $$\:S={S}_{W}+{S}_{B}=\sum\:_{g=1}^{G}{\pi\:}_{g}^{2}{S}_{g}+2\sum\:_{g=1}^{G}\sum\:_{h\ne\:g}{\pi\:}_{g}{\pi\:}_{h}{S}_{gh}$$

where $\:{\pi\:}_{g}$ is the share of country $\:g$ among the total number of deaths in the population, $\:{S}_{g}$ is the Simpson diversity index within country $\:g$ (i.e., it is the likelihood that two randomly chosen deaths within group $\:g$ are attributable to different causes), and $\:{S}_{gh}$ is the diversity between country $\:g$ and $\:h$ (i.e., it is the likelihood that a randomly chosen death from country $\:g$ and a randomly chosen death from country $\:h$ are attributable to different causes); details on how this decomposition formula arrives are shown in Additional file 1. The first term of the decomposition ($\:{S}_{W}$) is the within-country component, which is a weighted average of the amount of CoD diversity within the different country. The second term of the decomposition ($\:{S}_{B}$) is the between-country component, which measures how diverse mortality profiles are across all possible pairs of country. In our empirical example, the overall population will be the union of the populations of the 21 countries from Latin America and the Caribbean.

*Cause and country-specific contributions*

In empirical applications, it is useful to assess how much of the observed levels of CoD diversity are attributable to (i) a certain cause of death, (ii) a certain population subgroup, or (iii) a certain cause *and* population subgroup simultaneously. For that purpose, we use the equations shown below. First, one has that

3 $$\:S=\:\sum\:_{c=1}^{k}{p}_{c}(1-{p}_{c})=\sum\:_{c=1}^{k}{\mathcal{C}}_{c}$$

Thus, $\:{\mathcal{C}}_{c}={p}_{c}(1-{p}_{c})$ can be defined as the (absolute) contribution of cause of death $\:c$ to overall CoD diversity. Second, one has that

4 $$\:S=\sum\:_{g=1}^{G}\left[{\pi\:}_{g}^{2}{S}_{g}+{\pi\:}_{g}\sum\:_{h\ne\:g}{\pi\:}_{h}{S}_{gh}\right]=\sum\:_{g=1}^{G}\stackrel{\sim}{{\mathcal{C}}_{g}}$$

Thus, $\:\stackrel{\sim}{{\mathcal{C}}_{g}}={\pi\:}_{g}^{2}{S}_{g}+{\pi\:}_{g}\sum\:_{h\ne\:g}{\pi\:}_{h}{S}_{gh}$ can be defined as the contribution of country $\:g$ to the overall CoD diversity.

Last, by combining the previous cause- and country-specific decompositions simultaneously, one can see that the joint contribution of cause of death $\:c$ *and* country $\:g$ to overall CoD diversity can be defined as:

5 $$\:\stackrel{\sim}{{\mathcal{C}}_{c,g}}=\left[{\pi\:}_{g}^{2}\left({p}_{c,g}\left(1-{p}_{c,g}\right)\right)\right]+\left[{\pi\:}_{g}\left(1-{p}_{c,g}\right)\sum\:_{h\ne\:g}{\pi\:}_{h}{p}_{c,h}\right]$$

where $\:{p}_{c,g}$ is the share of deaths from cause $\:c$ within country $\:g$.

### Changing composition

As shown in Eq [2]., the values of $\:S$ and its changes over time depend not only on the within- country and between- country diversity terms (i.e., $\:{S}_{g}$, $\:{S}_{gh}$ in Eq [2]). but also on the levels and changes in the country’ population size (i.e., $\:{\pi\:}_{g}$). When other factors remain constant, more populated countries have greater impacts on overall diversity. To isolate the effect of shifting population compositions on changes in CoD diversity over time, we applied the method suggested by Kitagawa (1964). Formally, our CoD diversity indicator at time $\:t$ is denoted as $\:{S}_{t}=f({\varvec{\pi\:}}_{t},{\varvec{S}}_{t})$, that is, $\:S$ is a function of the vector of population shares (i.e., the different $\:{\pi\:}_{g}$) and the vector containing the within- country ($\:{S}_{g}$) and between- country ($\:{S}_{gh}$) diversity levels, all of which are measured in year $\:t$. With this notation, the changes in diversity over time can be decomposed as follows:

6 $$\:\varDelta\:S={S}_{2}-{S}_{1}=f\left({\varvec{\pi\:}}_{2},{\varvec{S}}_{2}\right)-f\left({\varvec{\pi\:}}_{1},{\varvec{S}}_{1}\right)={\varDelta\:}_{\pi\:}+{\varDelta\:}_{S}$$

where

$$\begin{aligned}&\:{\varDelta\:}_{\pi\:}\\&\quad=\frac{\left[\left(f\left({\varvec{\pi\:}}_{2},{\varvec{S}}_{2}\right)-f\left({\varvec{\pi\:}}_{1},{\varvec{S}}_{2}\right)\right)+\left(f\left({\varvec{\pi\:}}_{2},{\varvec{S}}_{1}\right)-f\left({\varvec{\pi\:}}_{1},{\varvec{S}}_{1}\right)\right)\right]}{2}\end{aligned}$$

$$\begin{aligned}&{\varDelta\:}_{S}\\&\quad=\frac{\left[\left(f\left({\varvec{\pi\:}}_{2},{\varvec{S}}_{2}\right)-f\left({\varvec{\pi\:}}_{2},{\varvec{S}}_{1}\right)\right)+\left(f\left({\varvec{\pi\:}}_{1},{\varvec{S}}_{2}\right)-f\left({\varvec{\pi\:}}_{1},{\varvec{S}}_{1}\right)\right)\right]}{2}\end{aligned}$$

The first term in Eq [6]. ($\:{\varDelta\:}_{\pi\:}$) can be interpreted as the contribution of compositional changes to changes in diversity, while the second term ($\:{\varDelta\:}_{S}$) measures the contribution of within- and between- country diversity changes to changes in overall diversity. This is a straightforward application of the decomposition method suggested by Kitagawa [22].

### Matrix notation

In a multigroup setting, the diversity index $\:S$ can be written using matrix notation as

7 $$\:S={\varvec{\pi\:}}^{\varvec{{\prime\:}}}\times\:{\mathcal{M}}_{S}\times\:\varvec{\pi\:}$$

where $\:\varvec{\pi\:}=({\pi\:}_{1},\cdots\:,{\pi\:}_{G})$ is the vector of population shares across countries, $\:\varvec{\pi\:}\varvec{{\prime\:}}$ denotes its transposed and

$$\mathcal{M}_S = \begin{pmatrix} S_1 & S_{12} & \cdots & S_{1G}\\[6pt] S_{21} & S_2 & \ddots & \vdots\\[6pt] \vdots & \ddots & \ddots & S_{G-1,G}\\[6pt] S_{G1} & \cdots & S_{G,G-1} & S_G \end{pmatrix}$$

is a symmetric matrix including the within- country diversity terms ($\:{S}_{g}$) in the diagonal and the between- country diversity terms ($\:{S}_{gh}$) off the diagonal. In our empirical example, this matrix has 21 rows and columns (one for each of the countries included in the analysis). The main advantage of matrix notation is that it allows for the visualization of each country’s contribution to the within and between components, independent of the effect of population shares $\:\pi\:$.

### Statistical inference

The uncertainty in the estimates is derived from the uncertainty associated with the proportion of deaths attributable to cause c in the total number of deaths, denoted as $\:\:{p}_{c}$​. If the total number of deaths due to cause c follows a binomial distribution:

$$\:{D}_{c}\:\sim\:Binomial(n,\:{p}_{c})$$

where $\:n$ represents the total number of deaths. In this context, the standard deviation of $\:\:{p}_{c}$​ is given by:

$$\:{SD(p}_{c})=\sqrt{\frac{{p}_{c}\left({1-p}_{c}\right)}{n}}$$

Given that the data are at the country level, $\:n$ is sufficiently large to assume that $\:\:{p}_{c}$​ follows a truncated normal distribution constrained between 0 and 1. Consequently, confidence intervals for the Simpson index of diversity and its components can be obtained through Monte Carlo simulations [2].

## Results

**CoD diversity trends**

**Fig. 1:**
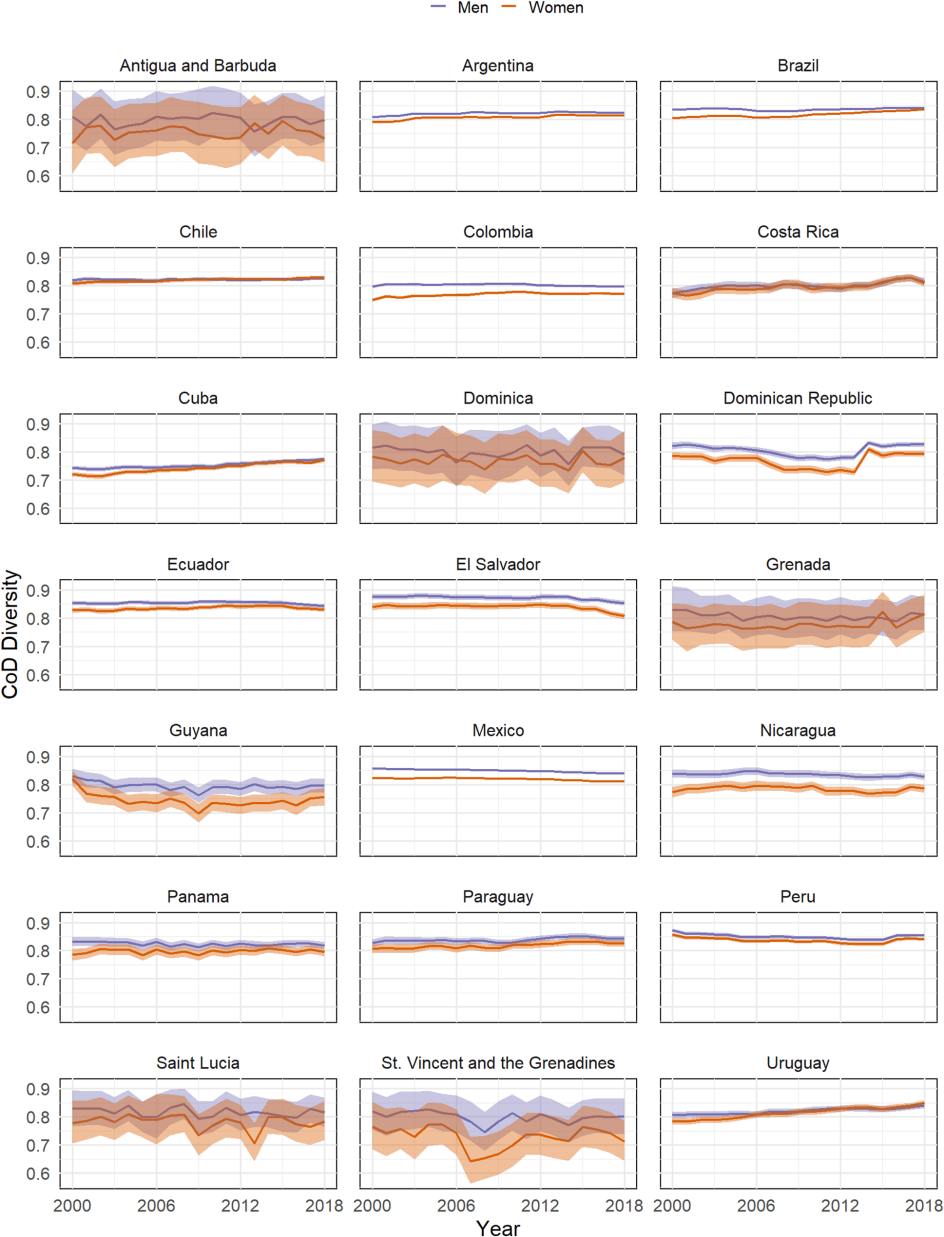
CoD diversity by Latin American and Caribbean countries (2000 to 2018). WHO Mortality Database [20] and World Population Prospects [21]

Latin American and Caribbean countries exhibit very different levels of CoD diversity over time (Fig. 1). In 2000, Antigua and Barbuda had the lowest diversity for women (0.717, CI: 0.610–0.835) and Cuba for men (0.744, CI: 0.737–0.751), while El Salvador and Peru were the most diverse for men (0.877 CI: 0.868–0.887) and women (0.857, CI: 0.851–0.864), respectively. In 2018, the countries with the least diversity were St. Vincent and the Grenadines among women (0.712, CI: 0.645–0.785) and Cuba among men (0.772, CI: 0.769–0.781). Moreover, Uruguay had the highest diversity among women (0.849, CI: 0.840–0.859), and Peru had the highest diversity among men (0.855, CI: 0.850–0.860). Generally, Caribbean countries had a lower level of diversity than Central and South American countries, but with noticeable fluctuations. In addition, CoD diversity tend to be higher among men across all countries, with statistically significant differences observed in Argentina, Brazil, Colombia, the Dominican Republic, Ecuador, El Salvador, Mexico, Nicaragua, and Peru.

Between 2000 and 2018, CoD diversity decreased in El Salvador, Mexico and Peru for both sexes and in Guyana for women. In contrast, it increased in Argentina, Brazil, Costa Rica, Cuba, and Uruguay for both sexes and in Chile and Colombia for women. The others countries did not show statistically significant changes over time.

**Fig. 2:**
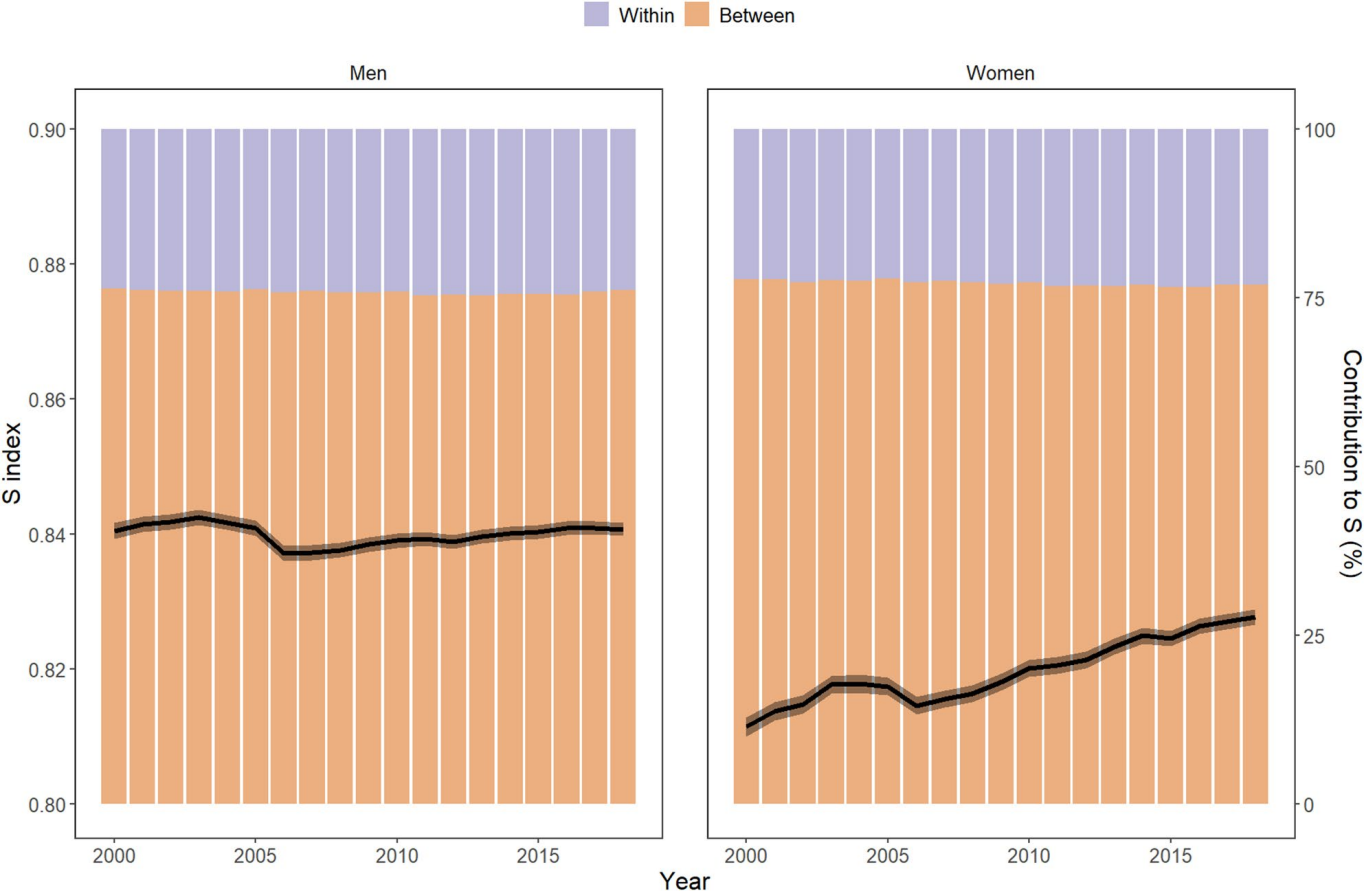
Overall CoD diversity in the LAC region and its components (2000–2018). WHO Mortality Database [20] and World Population Prospects [21]

Overall, the CoD diversity in the LAC region followed a nonmonotonic trend (Fig. 2). From 2000 to 2018, the CoD diversity increased from 0.811 (CI: 0.810–0.812) to 0.828 (CI: 0.828–0.829) for women and reached approximately 0.84 for men, with no statistically significant changes over time. Between these two years, the population composition of the region did not change substantially (Fig. 2A). Therefore, applying the Kitagawa decomposition method, we observed that changes in overall CoD diversity are mostly attributable to variations in the mortality profiles within countries (with changes in the death counts across countries playing a very minor role).

***CoD diversity decompositions***

**Fig. 3:**
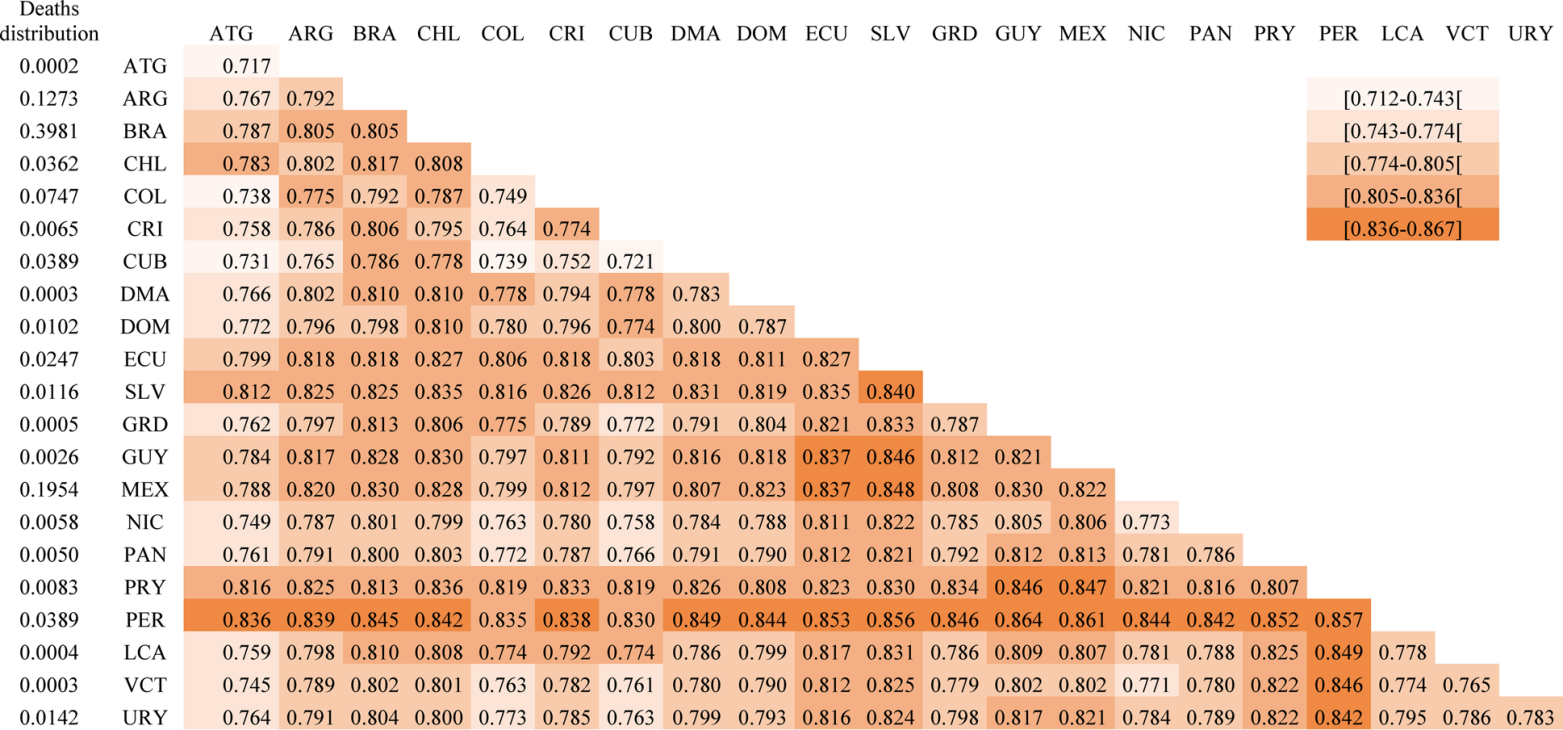
CoD diversity in multigroup settings – LACs countries, women (2000). WHO Mortality Database [20] and World Population Prospects [21]. Note: ATG - Antigua and Barbuda, ARG - Argentina, BRA - Brazil, CHL - Chile, COL - Colombia, CRI - Costa Rica, CUB - Cuba, DMA - Dominica, DOM - Dominican Republic, ECU - Ecuador, SLV - El Salvador, GRD - Grenada, GUY - Guyana, MEX - Mexico, NIC - Nicaragua, PAN - Panama, PRY - Paraguay, PER - Peru, LCA - Saint Lucia, VCT - Saint Vincent and Grenadines, URY - Uruguay

**Fig. 4:**
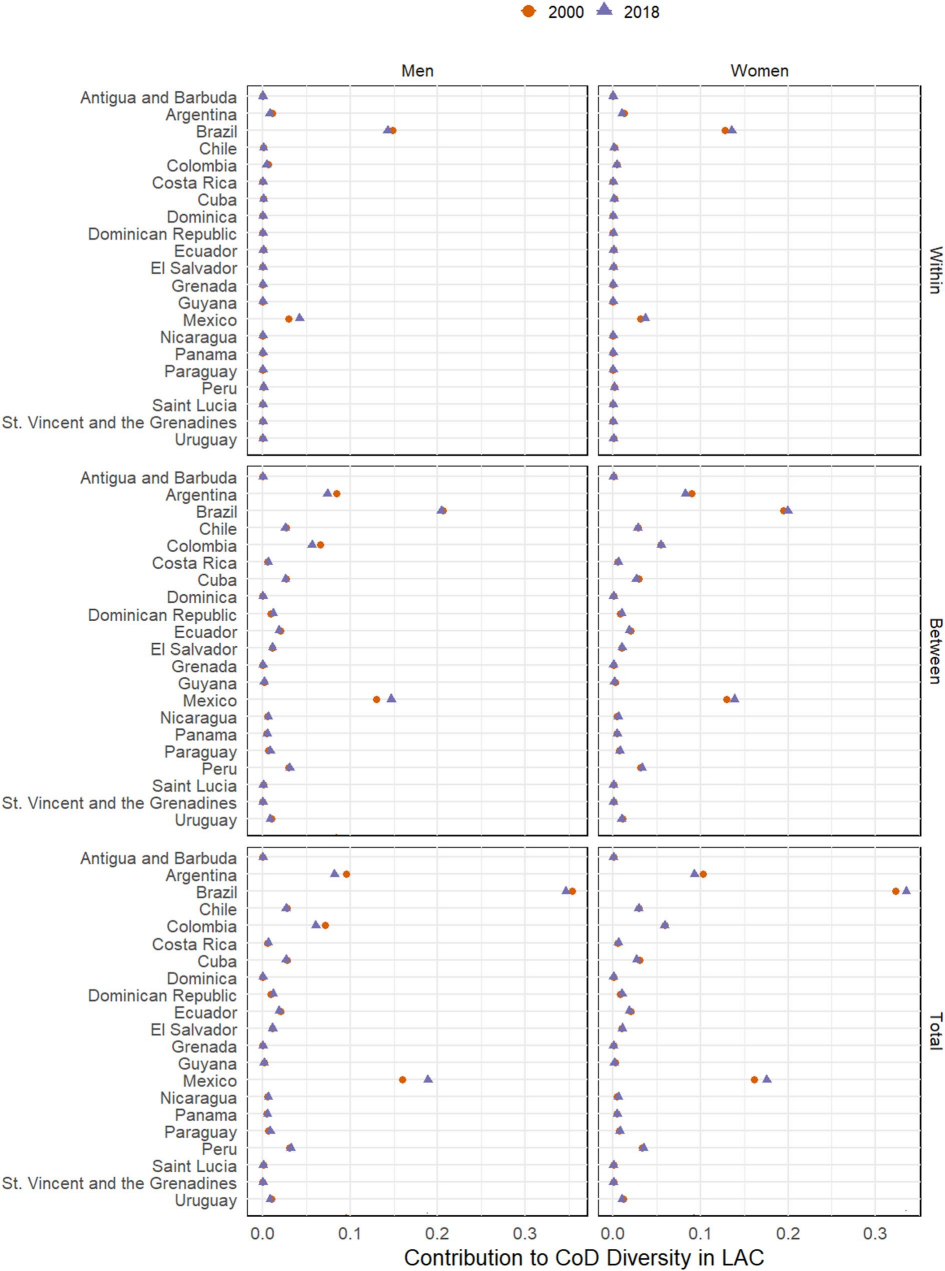
Countries’ contributions to CoD diversity in the LAC region and its components (2000 and 2018). WHO Mortality Database [20] and World Population Prospects [21].

**Fig. 5:**
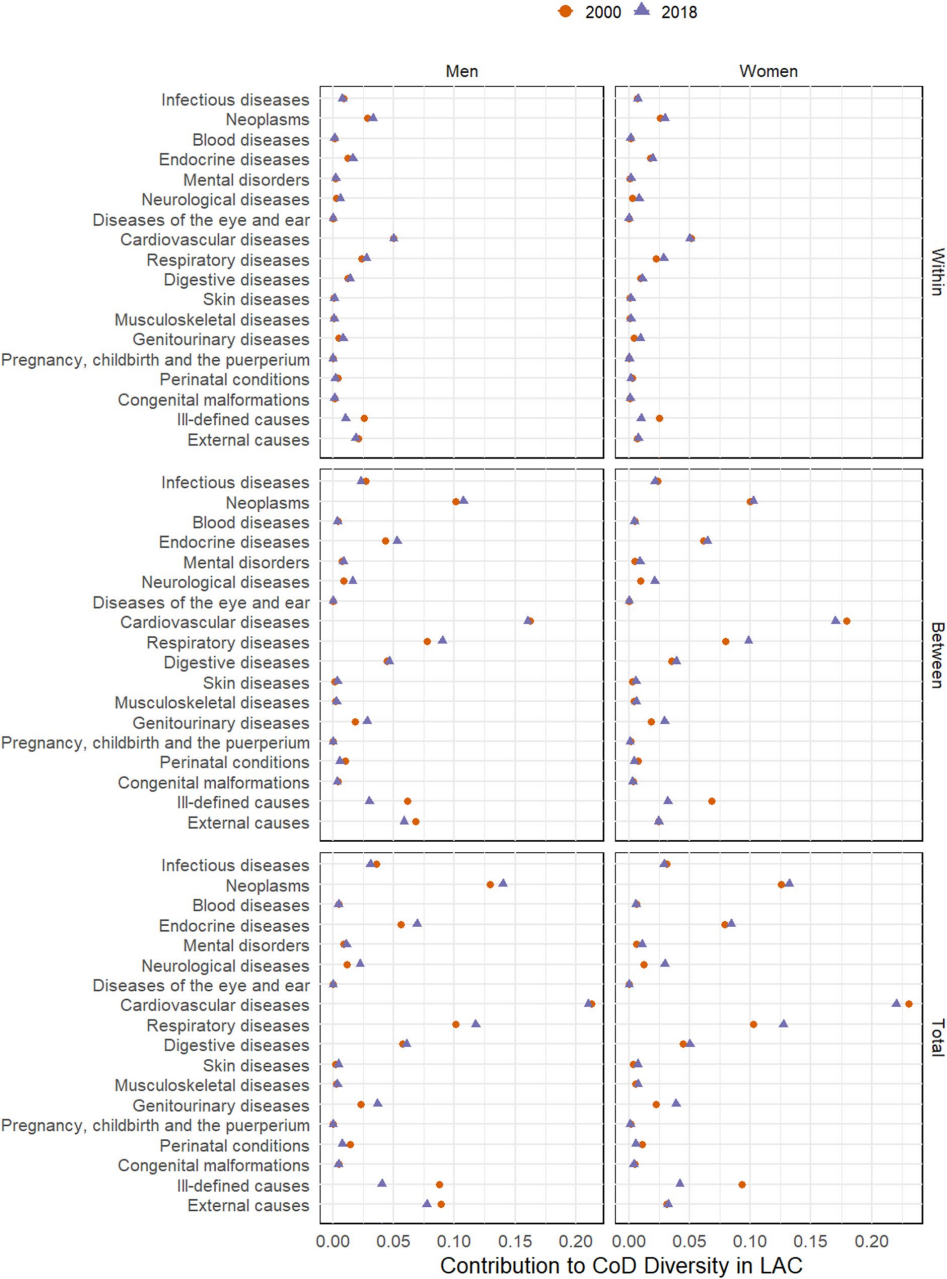
Causes of death contribution to CoD diversity in the LAC region and its components (2000 and 2018). WHO Mortality Database [20] and World Population Prospects [21]

As shown in the methods section, the overall CoD diversity levels in LACs can be broken down into several components. For instance, between 2000 and 2018, the differences in mortality profiles across countries accounted for approximately 77% of the overall CoD diversity in LACs, while diversity within countries explained only 23% (Fig. 2). This decomposition is further refined in Fig. 3, which shows all the within-group and between-group elements included in Eq. [2]. but displayed in matrix form (Eq. [3]). for women in the year 2000 (additional decompositions for the year 2018 and for men are available in Figs. 3A-5A). The elements in the main diagonal represent the within-country terms, reflecting the corresponding countries’ CoD diversity levels. Consequently, the results align with those presented in Fig. 1, with El Salvador and Peru having the most heterogeneous mortality profiles. On the other hand, the terms below the diagonal measure the differences between mortality profiles across all possible country pairs. Countries such as El Salvador, Mexico and, especially, Peru stand out because they exhibit the largest differences with respect to the other countries in the region.

***Countries’ contributions to CoD diversity***

Figure 4 (for confidence intervals see Table 1A) shows the contribution of the 21 LAC countries included in our analysis to overall CoD diversity and its within- and between-group components. Argentina, Brazil, Colombia, and Mexico (and, to a lesser extent, Chile and Peru) emerged as the major contributors to overall CoD diversity. The between component follows the same pattern as total CoD diversity, while Brazil and Mexico are the countries that contribute the most to within-country CoD diversity. Between 2000 and 2018, the contribution to overall CoD diversity decreased (statistically significant at 95%) in Argentina, Chile, Cuba, Ecuador, Guyana, and Uruguay for both sexes; in Brazil, Colombia, St. Vincent and the Grenadines for men; and in Grenada for women. On the other hand, it increased in Costa Rica, Dominican Republic, Mexico, Nicaragua, Panama, Paraguay and Peru for both sexes, for women in Brazil and El Salvador and for men in Grenada. Changes in contributions from other countries were not statically significant over time.

The correlation between the diversity of mortality profiles within each country and its contribution to overall CoD diversity was relatively low (although significant), at 0.23 for men and 0.28 for women (Fig. 5A).

***Causes of death contributing to CoD diversity***

We now show how the different causes of death have contributed to overall CoD diversity and its within- and between-country components (Fig. 5; Table 2 A). The main source of CoD diversity is cardiovascular mortality. In 2000, this cause contributed 0.213 units (25% of the total) to overall CoD diversity among men and 0.230 (28%) among women. In 2018, this cause contributed 0.210% (25%) to the CoD diversity among men and 0.220% (27%) among women. Notably, neoplasms, respiratory diseases, and external causes also expressively contribute to the diversity of CoD among men (Fig. 5). The distribution of CoD in both components (between and within) closely mirrors the overall CoD diversity in proportional terms.

Over the last century, communicable causes (infectious diseases, pregnancy, childbirth, and the puerperium, perinatal conditions and congenital malformations, deformations, and chromosomal abnormalities) have played a crucial role in increasing life expectancy and reducing variation in age at death, as there has been a substantial reduction in mortality from these causes in younger age groups [8, 10, 23]. On the other hand, even if there was a statistically significant reduction in the contribution of communicable causes to overall CoD diversity from 2000 to 2018, this effect was small since these causes currently contribute little to the total mortality of the population.

In addition to communicable causes, cardiovascular diseases decreased their participation, while other noncommunicable causes (neoplasms, endocrine diseases, mental disorders, neurological diseases, respiratory diseases, digestive diseases, skin diseases, musculoskeletal diseases and genitourinary diseases) increased their participation in overall CoD diversity over time. In contrast, the contribution of external causes decreased among men and does not present statistically significant changes for women in the same period. Finally, the contribution of ill-defined causes decreased from approximately 10% in 2000 to 5% in 2018, reflecting improvements in the quality and completeness of mortality information.

It is also important to note that the correlation between the proportion of deaths from a specific cause and its contribution to overall CoD diversity is low (Supplementary Fig. 6A). Despite cardiovascular diseases being the most prevalent in the mortality profiles across all countries, the contribution of this cause can vary considerably from one country to another.

## Discussion

The CoD profile has been employed as a key marker of the health and well-being of populations through the perspective of epidemiological transition theory. However, using CoD diversity indicators and their determinants has only recently become noteworthy [1–3]. In this paper, we propose a novel decomposition method that allows the identification of the main sources of CoD diversity that can be applied in different settings where the population under study is divided across groups (e.g., socioeconomic status, race, gender, or any other sociodemographic groups). This approach makes it possible to identify the sources of health disparities in a population, providing valuable insights into how social, economic, environmental, and behavioral factors impact population health and, consequently, CoD diversity. In this way, analysts and policy-makers can assess whether some groups contribute more or less to the uncertainty around the causes from which individuals die—a useful tool for the design of policies aimed at identifying and reducing health inequalities.

To illustrate the usefulness of the suggested approach, we applied it to mortality profiles from Latin America and the Caribbean, which are among the regions with the greatest socioeconomic and health inequalities in the world. These inequalities, in turn, translate into high heterogeneity in life expectancy and causes of death between and within countries [13, 17]. Calazans and Permanyer [2] highlighted that the LAC region has a high diversity of causes of death, mainly due to overlapping phases in the epidemiological transition process. Previous studies have shown that some countries face difficulties in completing phases of the epidemiological transition due to poverty, precarious living conditions and limited access to public health services [8, 15, 16]. In this way, it is possible to observe a high mortality rate from communicable causes simultaneously with high mortality rates from noncommunicable diseases, especially cardiovascular diseases, which therefore translates into a high diversity in causes of death.

In addition, there is a significant variability in CoD diversity among LAC countries. Although all LAC countries have CoD profiles with a high prevalence of chronic diseases, the concentration around these causes varies significantly between countries. For example, among women in the year 2000, the proportion of deaths due to cardiovascular diseases (the most predominant cause of death) ranged from 23% in Peru (with the highest diversity at 0.857) to 48% in Antigua and Barbuda (with the lowest diversity at 0.717).

Cardiovascular deaths are not only the main contributor to overall CoD diversity levels in the region but also the most important factor driving changes in CoD diversity over time. As cardiovascular deaths decline, they give way to other chronic causes, which become more prominent and contribute to diversifying the corresponding mortality profiles. This result had already been emphasized by Calazans & Permanyer [2], who indicated that the LAC region has already initiated, to some extent, the so-called ‘cardiovascular revolution’ [24]. We must also highlight the importance of external causes, driven by violent deaths and traffic accidents, in the formation of complex and uneven epidemiological profiles of the Americas, especially Brazil, Colombia, Ecuador, El Salvador, Mexico and Venezuela [15, 16, 25–28]. Such deaths typically affect men more often than women – a phenomenon that makes the mortality profiles of men more diverse than those of women.

It is interesting to note that El Salvador, Peru, and Uruguay are the countries that contribute the most to explaining the differences in the mortality profile between countries in the region, but for very different, and opposed, reasons. On the one hand, the high CoD diversity (and therefore the high contribution) in El Salvador and Peru can be explained by expressive participation of causes of death related to the early stages of the epidemiological transition, such as communicable causes, respiratory causes, and external causes. On the other hand, the high CoD diversity in Uruguay is due to a mortality profile characteristic of later stages of the transition, with higher prevalence of deaths attributable to chronic conditions. This finding is corroborated by previous studies that highlighted the complexity of the epidemiological transition process in the LAC region, which is deeply marked by cycles of convergence and divergence between countries [15, 24, 29, 30].

These findings are crucial for shaping public health policies in the region, as variations in CoD diversity reflect a complex interplay of socioeconomic, political, historical, and cultural factors that influence morbidity and mortality profiles. Even if chronic diseases are the leading causes of mortality, the concentration of these causes varies significantly between countries. Thus, policies must consider the unique mortality profile of each country, as strategies that are effective in reducing mortality in one context may not yield the same results in another. Additionally, it should be noted that countries with greater CoD diversity often exhibit higher unpredictability in their mortality profiles, thus rendering disease diagnoses more complex and error-prone and posing additional challenges for healthcare systems. Acknowledging this unpredictability enables policymakers to craft compelling, context-specific strategies aimed at reducing mortality and improving health outcomes throughout the region.

This study is subject to certain limitations. The first limitation relates to the availability and quality of mortality data in Latin America and the Caribbean. Although the coverage of death records has improved in recent decades in the region, the data quality is still limited. Thus, we choose a set of 21 countries with longer and more consistent historical series to represent the region. Although these countries represent more than 85% of deaths in the region, we are aware that some countries with high mortality rates (such as Venezuela and Haiti) are excluded from the analysis. The coverage of the death registry is, in turn, corrected when the mortality rates estimated by the World Population Prospect are used. The quality of cause-of-death information can also introduce bias into the CoD diversity indicator. In countries where the quality of this information is low, deaths tend to be more concentrated around ill-defined causes, thereby affecting our estimates. However, only two countries (El Salvador in 2013 and 2014, and Paraguay in 2000, 2002, and 2003) exhibit low-quality cause-of-death information, according to the criterion proposed by Mathers et al. [31], which considers death records to be of low quality when the proportion of ill-defined causes exceeds 20%. Besides, this study, like other cross-country analyses of cause-of-death patterns, may face additional problems due to variations in the accuracy of cause-of-death diagnoses across countries. To minimize this issue, we rely on broad ICD-10 chapters rather than specific causes of death, which are more prone to comparability problems.

The second limitation pertains to the diversity indicators. On the one hand, diversity indicators are sensitive to the number of categories with which one works [32, 33]. To reduce this problem, we used the same CoD grouping across countries during the entire period. On the other hand, these indicators are also susceptible to small numbers; therefore, the Caribbean islands’ time series present many oscillations. Third, the large number of countries used in the decomposition makes the analysis very rich but could complicate the interpretation of the findings. In addition, when the number of groups becomes very large, the between-group component explains almost all the observed variability. This “mechanical” relationship between the number of groups and the relevance of the between-group component is unavoidable and occurs as well in all metrics admitting similar decompositions (see, for example, Permanyer, Sasson and Villavicencio [34]). The last limitation concerns the demographic and epidemiological changes in the mortality profile. As mortality increasingly shifts to more advanced conditions, the presence of comorbidities becomes progressively more prevalent, thus complicating the attribution of an only underlying cause of death [35–38]. Future research should adopt more comprehensive multiple causes of death approaches in CoD diversity analysis, as we can see in Trias-Llimós and Permanyer [3]. Despite these limitations, the method proposed in this paper to decompose CoD diversity should contribute to a better understanding of contemporary mortality dynamics, especially in a context with large health inequalities.

## Supplementary Information

Supplementary Material 1.

## Data Availability

We used information from the Mortality Database by the World Health Organization and from World Population Prospects. These two data sources are freely accessible, do not require prior registration to use and were referenced in the manuscript, respectively as: World Health Organization. Mortality Database. Available in: https://www.who.int/data/data-collection-tools/who-mortality-database (accessed May 15, 2023)United Nations, Department of Economic and Social Affairs, Population Division (2022). World Population Prospects 2022, Online Edition.The scripts generated during the current study are available in the Git-hub repository (the link will be made available after the manuscript has been evaluated). We conducted our analyses using the open-source statistical software R (version R-4.1.0).

## Abbreviations

CoD: Causes of death
LAC: Latin America and the Caribbean
