# Supplementary material for: Cause of death diversity in multi-group settings: an application to Latin America and the Caribbean
Source: Popul Health Metr. 2025 Dec 8;24:1. doi: 10.1186/s12963-025-00436-3 (PMC12797717; doi:10.1186/s12963-025-00436-3)
Supplement: Supplementary file 1 — Supplementary Material 1. [file 12963_2025_436_MOESM1_ESM.docx]

# **ADDITIONAL FILES - CAUSE OF DEATH DIVERSITY IN MULTI-GROUP SETTINGS: AN APPLICATION TO LATIN AMERICA AND THE CARIBBEAN**

# **Additional file 1: Derivation of the formula for decomposition into between-group and within-group components**

In this paper, CoD diversity is measured using the Simpson index of diversity, $S$. Assuming that all deaths are classified into a list of $k$ mutually exclusive causes, $S$ is defined as the probability that two randomly chosen deaths are attributable to different causes. Formally, it is defined as

$$S=1- \sum_{c=1}^{k} p_{c}^{2}$$

where $p_{c}$ is the share of deaths attributable to cause $c$ among all deaths.

Assume our population is composed of $G$ countries. When this happens, $p_{c}$can be written as the sum of share of deaths attributable to cause *c* within countrt *g* ($p_{c,g}$) weighted by the share of country $g$ among the total number of deaths in the population ($\pi_{g})$. Formally:

$$p_{c}= \pi_{A}p_{c,A}+\pi_{B}p_{c,B}+ \pi_{C}p_{c,C}+ \ldots{+ \pi}_{G}p_{c,G}$$

Thus,

$$S=1- \sum_{c=1}^{k} {(\pi_{A}p_{c,A}+\pi_{B}p_{c,B}+ \pi_{C}p_{c,C}+ \ldots{+ \pi}_{G}p_{c,G})}^{2}$$

$$S=1- \sum_{c=1}^{k} \left[ \left( \sum_{g=1}^{G} \pi_{g}^{2}p_{c,g}^{2} \right)+ \left( 2\sum_{g=1}^{G} \sum_{h \neq g} \pi_{g}p_{c,g}\pi_{h}p_{c,h} \right) \right]$$

$$S=1- \left[ \left( \sum_{g=1}^{G} \pi_{g}^{2}\sum_{c=1}^{k} p_{c,g}^{2} \right)+\left( 2\sum_{g=1}^{G} \sum_{h \neq g} \pi_{g}\pi_{h}\sum p_{c,g}p_{c,h} \right) \right]$$

By definition, the different country shares $\pi_{g}$ add up to one, so one has that:

$$\pi_{A}+\pi_{B}+ \pi_{C}+ \ldots{+ \pi}_{G}=1$$

$${{(\pi}_{A}+\pi_{B}+ \pi_{C}+ \ldots{+ \pi}_{G})}^{2}=1$$

$$\left[ \left( \sum_{g=1}^{G} \pi_{g}^{2} \right)+\left( 2\sum_{g=1}^{G} \sum_{h \neq g} \pi_{g}\pi_{h} \right) \right]=1$$

Thus,

$$S=\left[ \left( \sum_{g=1}^{G} \pi_{g}^{2} \right)+\left( 2\sum_{g=1}^{G} \sum_{h \neq g} \pi_{g}\pi_{h} \right) \right] - \left[ \left( \sum_{g=1}^{G} \pi_{g}^{2}\sum_{c=1}^{k} p_{c,g}^{2} \right)+\left( 2\sum_{g=1}^{G} \sum_{h \neq g} \pi_{g}\pi_{h}\sum_{c=1}^{k} p_{c,g}p_{c,h} \right) \right]$$

$$S=\left[ \left( \sum_{g=1}^{G} \pi_{g}^{2} \right)-\left( \sum_{g=1}^{G} \pi_{g}^{2}\sum_{c=1}^{k} p_{c,g}^{2} \right) \right] + \left[ \left( 2\sum_{g=1}^{G} \sum_{h \neq g} \pi_{g}\pi_{h} \right)-\left( 2\sum_{g=1}^{G} \sum_{h \neq g} \pi_{g}\pi_{h}\sum_{c=1}^{k} p_{c,g}p_{c,h} \right) \right]$$

$$S=\left[ \left( \sum_{g=1}^{G} \pi_{g}^{2} \right)\left( 1-\sum_{c=1}^{k} p_{c,g}^{2} \right) \right] + \left[ \left( 2\sum_{g=1}^{G} \sum_{h \neq g} \pi_{g}\pi_{h} \right)\left( 1- \sum_{c=1}^{k} p_{c,g}p_{c,h} \right) \right]$$

$$S=\left[ \left( \sum_{g=1}^{G} \pi_{g}^{2} \right)S_{g} \right] + \left[ \left( 2\sum_{g=1}^{G} \sum_{h \neq g} \pi_{g}\pi_{h} \right)S_{gh} \right]$$

$$S=S_{W}+S_{B}=\sum_{g=1}^{G} \pi_{g}^{2}S_{g}+2\sum_{g=1}^{G} \sum_{h\neq g} \pi_{g}\pi_{h}S_{gh}$$

This is the decomposition we were looking for. $S_{W}$ is the within-country component, which is a weighted average of the amount of CoD diversity within the different countries, and $S_{B}$ is the between-country component, which measures how diverse mortality profiles are across all possible pairs of countries.

In the particular case when the number of countries ($G$) equals 2, the previous formulas simplify considerably. They would look like this:

$$S=S_{W}+S_{B}=\pi_{1}^{2}S_{1}+\pi_{2}^{2}S_{2}+2\pi_{1}\pi_{2}S_{12}$$

where $\pi_{1}, \pi_{2}$ are the population shares of countries 1 and 2, and

$$S_{1}=1-\sum_{c=1}^{k} p_{c,1}^{2}$$

$$S_{2}=1-\sum_{c=1}^{k} p_{c,2}^{2}$$

$$S_{12}=1- \sum_{c=1}^{k} p_{c,1}p_{c,2}$$

# **Additional file 2: Additional figures and tables**

# **Figure 1A – Distribution of deaths by cause in the LAC region (2000 to 2018)**


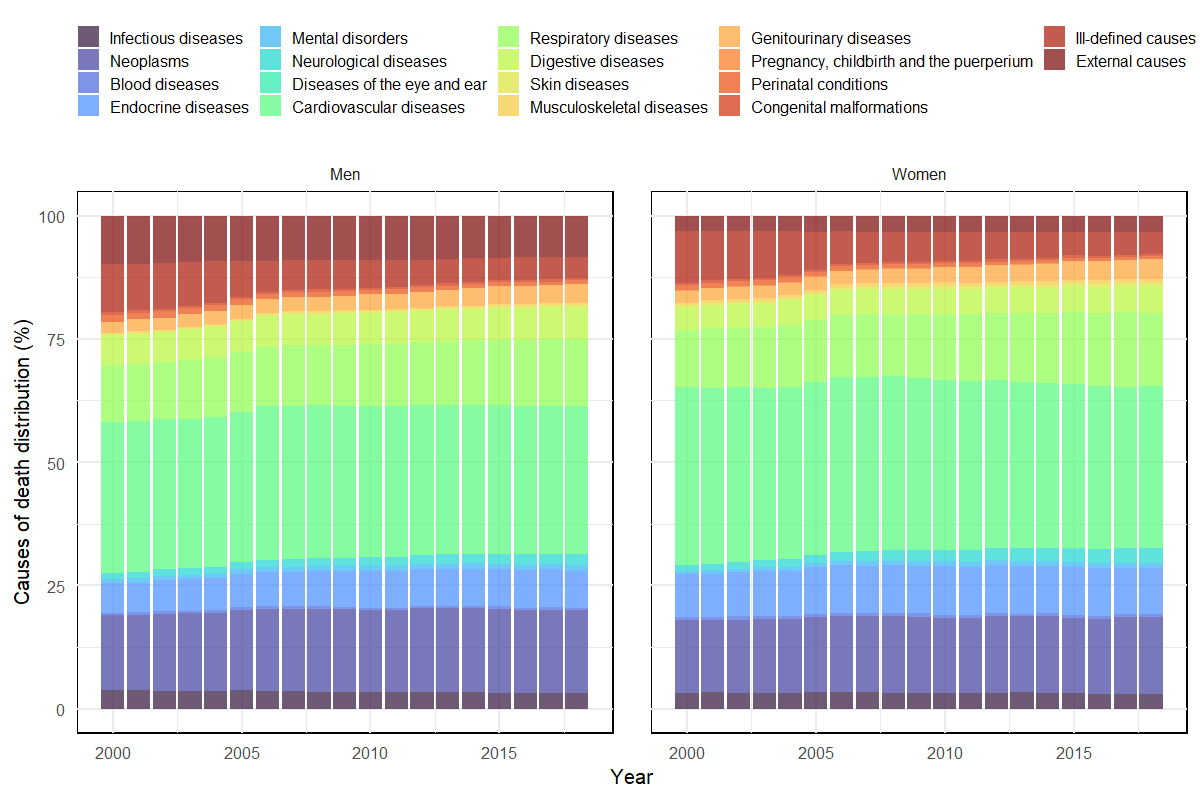


Source: WHO Mortality Database [20] and World Population Prospects [21].

# **Figure 2A – Distribution of deaths by country in the LAC region (2000 to 2018)**


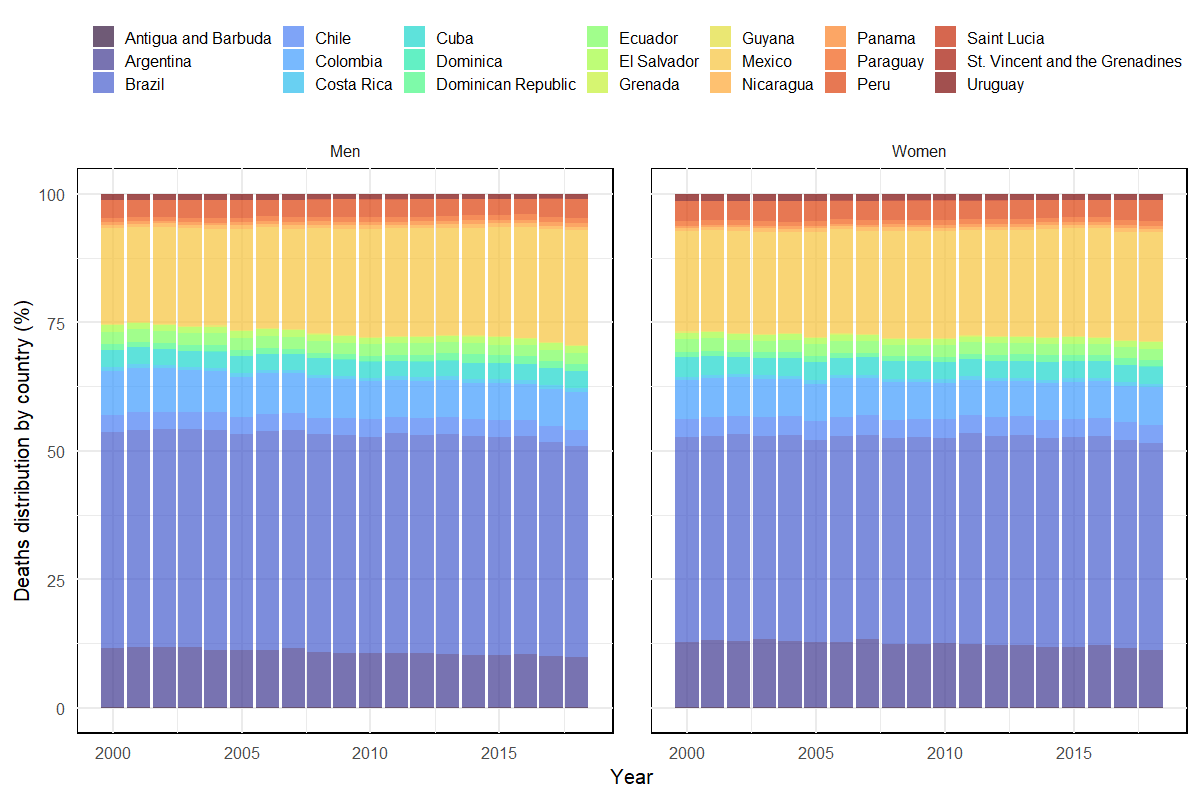


Source: WHO Mortality Database [20] and World Population Prospects [21].

# **Figure 3A - CoD diversity in multigroup settings – LACs countries, women (2018)**

| Deaths distribution |  | ATG | ARG | BRA | CHL | COL | CRI | CUB | DMA | DOM | ECU | SLV | GRD | GUY | MEX | NIC | PAN | PRY | PER | LCA | VCT | URY |
| --- | --- | --- | --- | --- | --- | --- | --- | --- | --- | --- | --- | --- | --- | --- | --- | --- | --- | --- | --- | --- | --- | --- |
| 0.0002 | ATG | 0.734 |  |  |  |  |  |  |  |  |  |  |  |  |  |  |  |  |  |  |  |  |
| 0.1123 | ARG | 0.802 | 0.817 |  |  |  |  |  |  |  |  |  |  |  |  |  |  |  | [0.712-0.743[ | | |  |
| 0.4024 | BRA | 0.803 | 0.830 | 0.836 |  |  |  |  |  |  |  |  |  |  |  |  |  |  | [0.743-0.774[ | | |  |
| 0.0350 | CHL | 0.807 | 0.829 | 0.836 | 0.830 |  |  |  |  |  |  |  |  |  |  |  |  |  | [0.774-0.805[ | | |  |
| 0.0733 | COL | 0.763 | 0.801 | 0.810 | 0.807 | 0.771 |  |  |  |  |  |  |  |  |  |  |  |  | [0.805-0.836[ | | |  |
| 0.0070 | CRI | 0.788 | 0.822 | 0.827 | 0.822 | 0.794 | 0.810 |  |  |  |  |  |  |  |  |  |  |  | [0.836-0.867] | | |  |
| 0.0333 | CUB | 0.770 | 0.808 | 0.815 | 0.810 | 0.776 | 0.797 | 0.772 |  |  |  |  |  |  |  |  |  |  |  |  |  |  |
| 0.0002 | DMA | 0.761 | 0.815 | 0.818 | 0.817 | 0.780 | 0.801 | 0.786 | 0.779 |  |  |  |  |  |  |  |  |  |  |  |  |  |
| 0.0124 | DOM | 0.771 | 0.815 | 0.820 | 0.825 | 0.788 | 0.812 | 0.793 | 0.792 | 0.793 |  |  |  |  |  |  |  |  |  |  |  |  |
| 0.0224 | ECU | 0.798 | 0.830 | 0.835 | 0.835 | 0.808 | 0.824 | 0.814 | 0.814 | 0.818 | 0.830 |  |  |  |  |  |  |  |  |  |  |  |
| 0.0124 | SLV | 0.799 | 0.828 | 0.836 | 0.842 | 0.812 | 0.834 | 0.819 | 0.818 | 0.811 | 0.829 | 0.807 |  |  |  |  |  |  |  |  |  |  |
| 0.0003 | GRD | 0.783 | 0.832 | 0.832 | 0.834 | 0.804 | 0.820 | 0.809 | 0.803 | 0.812 | 0.828 | 0.836 | 0.815 |  |  |  |  |  |  |  |  |  |
| 0.0015 | GUY | 0.747 | 0.810 | 0.811 | 0.815 | 0.773 | 0.796 | 0.778 | 0.772 | 0.779 | 0.807 | 0.808 | 0.794 | 0.756 |  |  |  |  |  |  |  |  |
| 0.2122 | MEX | 0.782 | 0.833 | 0.833 | 0.837 | 0.805 | 0.821 | 0.813 | 0.804 | 0.812 | 0.828 | 0.834 | 0.817 | 0.792 | 0.811 |  |  |  |  |  |  |  |
| 0.0071 | NIC | 0.764 | 0.818 | 0.819 | 0.822 | 0.785 | 0.806 | 0.792 | 0.786 | 0.794 | 0.815 | 0.815 | 0.805 | 0.774 | 0.802 | 0.786 |  |  |  |  |  |  |
| 0.0058 | PAN | 0.770 | 0.817 | 0.820 | 0.821 | 0.787 | 0.807 | 0.792 | 0.790 | 0.797 | 0.817 | 0.817 | 0.810 | 0.780 | 0.810 | 0.792 | 0.795 |  |  |  |  |  |
| 0.0094 | PRY | 0.793 | 0.832 | 0.835 | 0.838 | 0.810 | 0.827 | 0.815 | 0.813 | 0.815 | 0.830 | 0.826 | 0.825 | 0.803 | 0.825 | 0.811 | 0.815 | 0.826 |  |  |  |  |
| 0.0407 | PER | 0.840 | 0.839 | 0.852 | 0.849 | 0.831 | 0.844 | 0.840 | 0.846 | 0.850 | 0.853 | 0.867 | 0.854 | 0.847 | 0.855 | 0.850 | 0.849 | 0.859 | 0.840 |  |  |  |
| 0.0003 | LCA | 0.763 | 0.823 | 0.822 | 0.823 | 0.788 | 0.806 | 0.794 | 0.786 | 0.797 | 0.816 | 0.821 | 0.802 | 0.774 | 0.801 | 0.787 | 0.793 | 0.811 | 0.851 | 0.784 |  |  |
| 0.0003 | VCT | 0.725 | 0.801 | 0.799 | 0.804 | 0.758 | 0.783 | 0.763 | 0.753 | 0.764 | 0.794 | 0.791 | 0.779 | 0.738 | 0.778 | 0.757 | 0.762 | 0.788 | 0.846 | 0.756 | 0.712 |  |
| 0.0116 | URY | 0.826 | 0.844 | 0.851 | 0.845 | 0.828 | 0.840 | 0.825 | 0.836 | 0.839 | 0.847 | 0.844 | 0.850 | 0.834 | 0.855 | 0.840 | 0.837 | 0.848 | 0.867 | 0.841 | 0.822 | 0.849 |

Source: WHO Mortality Database [20] and World Population Prospects [21]. Note: ATG - Antigua and Barbuda, ARG - Argentina, BRA - Brazil, CHL - Chile, COL - Colombia, CRI - Costa Rica, CUB - Cuba, DMA - Dominica, DOM - Dominican Republic, ECU - Ecuador, SLV - El Salvador, GRD - Grenada, GUY - Guyana, MEX - Mexico, NIC - Nicaragua, PAN - Panama, PRY - Paraguay, PER - Peru, LCA - Saint Lucia, VCT - Saint Vincent and Grenadines, URY - Uruguay.

# **Figure 4A - CoD diversity in multigroup settings– LACs countries, men (2000)**

| Deaths distribution |  | ATG | ARG | BRA | CHL | COL | CRI | CUB | DMA | DOM | ECU | SLV | GRD | GUY | MEX | NIC | PAN | PRY | PER | LCA | VCT | URY |
| --- | --- | --- | --- | --- | --- | --- | --- | --- | --- | --- | --- | --- | --- | --- | --- | --- | --- | --- | --- | --- | --- | --- |
| 0.0002 | ATG | 0.811 |  |  |  |  |  |  |  |  |  |  |  |  |  |  |  |  |  |  |  |  |
| 0.1152 | ARG | 0.818 | 0.809 |  |  |  |  |  |  |  |  |  |  |  |  |  |  |  | [0.744-0.773[ | | |  |
| 0.4211 | BRA | 0.849 | 0.830 | 0.835 |  |  |  |  |  |  |  |  |  |  |  |  |  |  | [0.773-0.802[ | | |  |
| 0.0327 | CHL | 0.819 | 0.817 | 0.840 | 0.819 |  |  |  |  |  |  |  |  |  |  |  |  |  | [0.802-0.831[ | | |  |
| 0.0864 | COL | 0.824 | 0.812 | 0.831 | 0.818 | 0.797 |  |  |  |  |  |  |  |  |  |  |  |  | [0.831-0.860[ | | |  |
| 0.0065 | CRI | 0.801 | 0.795 | 0.823 | 0.801 | 0.792 | 0.774 |  |  |  |  |  |  |  |  |  |  |  | [0.860-0.889] | | |  |
| 0.0343 | CUB | 0.795 | 0.783 | 0.813 | 0.791 | 0.781 | 0.761 | 0.744 |  |  |  |  |  |  |  |  |  |  |  |  |  |  |
| 0.0002 | DMA | 0.825 | 0.817 | 0.837 | 0.826 | 0.820 | 0.803 | 0.792 | 0.815 |  |  |  |  |  |  |  |  |  |  |  |  |  |
| 0.0106 | DOM | 0.838 | 0.822 | 0.831 | 0.833 | 0.820 | 0.812 | 0.802 | 0.828 | 0.822 |  |  |  |  |  |  |  |  |  |  |  |  |
| 0.0243 | ECU | 0.855 | 0.841 | 0.847 | 0.849 | 0.839 | 0.834 | 0.826 | 0.846 | 0.841 | 0.854 |  |  |  |  |  |  |  |  |  |  |  |
| 0.0127 | SLV | 0.882 | 0.867 | 0.867 | 0.873 | 0.858 | 0.861 | 0.855 | 0.874 | 0.863 | 0.872 | 0.877 |  |  |  |  |  |  |  |  |  |  |
| 0.0002 | GRD | 0.833 | 0.830 | 0.851 | 0.838 | 0.830 | 0.816 | 0.808 | 0.826 | 0.842 | 0.857 | 0.881 | 0.831 |  |  |  |  |  |  |  |  |  |
| 0.0020 | GUY | 0.843 | 0.833 | 0.849 | 0.842 | 0.822 | 0.818 | 0.809 | 0.833 | 0.837 | 0.854 | 0.871 | 0.837 | 0.830 |  |  |  |  |  |  |  |  |
| 0.1862 | MEX | 0.848 | 0.846 | 0.861 | 0.849 | 0.840 | 0.832 | 0.826 | 0.845 | 0.855 | 0.866 | 0.884 | 0.848 | 0.852 | 0.857 |  |  |  |  |  |  |  |
| 0.0060 | NIC | 0.841 | 0.832 | 0.847 | 0.839 | 0.824 | 0.816 | 0.808 | 0.834 | 0.837 | 0.853 | 0.870 | 0.842 | 0.840 | 0.854 | 0.839 |  |  |  |  |  |  |
| 0.0053 | PAN | 0.832 | 0.823 | 0.838 | 0.831 | 0.823 | 0.812 | 0.803 | 0.830 | 0.830 | 0.847 | 0.870 | 0.841 | 0.842 | 0.855 | 0.841 | 0.832 |  |  |  |  |  |
| 0.0079 | PRY | 0.859 | 0.838 | 0.835 | 0.849 | 0.842 | 0.836 | 0.828 | 0.845 | 0.832 | 0.846 | 0.866 | 0.861 | 0.859 | 0.870 | 0.854 | 0.842 | 0.827 |  |  |  |  |
| 0.0350 | PER | 0.863 | 0.856 | 0.864 | 0.859 | 0.861 | 0.852 | 0.846 | 0.865 | 0.864 | 0.872 | 0.886 | 0.874 | 0.875 | 0.879 | 0.874 | 0.865 | 0.867 | 0.873 |  |  |  |
| 0.0003 | LCA | 0.828 | 0.823 | 0.840 | 0.828 | 0.821 | 0.810 | 0.801 | 0.829 | 0.831 | 0.848 | 0.871 | 0.839 | 0.841 | 0.851 | 0.840 | 0.832 | 0.845 | 0.866 | 0.829 |  |  |
| 0.0003 | VCT | 0.820 | 0.820 | 0.843 | 0.826 | 0.820 | 0.804 | 0.796 | 0.822 | 0.831 | 0.850 | 0.877 | 0.830 | 0.836 | 0.848 | 0.837 | 0.831 | 0.851 | 0.868 | 0.828 | 0.820 |  |
| 0.0125 | URY | 0.815 | 0.811 | 0.833 | 0.814 | 0.814 | 0.796 | 0.786 | 0.823 | 0.824 | 0.843 | 0.869 | 0.837 | 0.841 | 0.850 | 0.835 | 0.823 | 0.838 | 0.857 | 0.822 | 0.820 | 0.806 |

Source: WHO Mortality Database [20] and World Population Prospects [21]. Note: ATG - Antigua and Barbuda, ARG - Argentina, BRA - Brazil, CHL - Chile, COL - Colombia, CRI - Costa Rica, CUB - Cuba, DMA - Dominica, DOM - Dominican Republic, ECU - Ecuador, SLV - El Salvador, GRD - Grenada, GUY - Guyana, MEX - Mexico, NIC - Nicaragua, PAN - Panama, PRY - Paraguay, PER - Peru, LCA - Saint Lucia, VCT - Saint Vincent and Grenadines, URY - Uruguay.

# **Figure 5A - CoD diversity in multigroup settings – LACs countries, men (2018)**

| Deaths distribution |  | ATG | ARG | BRA | CHL | COL | CRI | CUB | DMA | DOM | ECU | SLV | GRD | GUY | MEX | NIC | PAN | PRY | PER | LCA | VCT | URY |
| --- | --- | --- | --- | --- | --- | --- | --- | --- | --- | --- | --- | --- | --- | --- | --- | --- | --- | --- | --- | --- | --- | --- |
| 0.0002 | ATG | 0.800 |  |  |  |  |  |  |  |  |  |  |  |  |  |  |  |  |  |  |  |  |
| 0.0977 | ARG | 0.831 | 0.826 |  |  |  |  |  |  |  |  |  |  |  |  |  |  |  | [0.744-0.773[ | | |  |
| 0.4115 | BRA | 0.833 | 0.837 | 0.842 |  |  |  |  |  |  |  |  |  |  |  |  |  |  | [0.773-0.802[ | | |  |
| 0.0315 | CHL | 0.827 | 0.831 | 0.839 | 0.827 |  |  |  |  |  |  |  |  |  |  |  |  |  | [0.802-0.831[ | | |  |
| 0.0733 | COL | 0.810 | 0.818 | 0.823 | 0.819 | 0.797 |  |  |  |  |  |  |  |  |  |  |  |  | [0.831-0.860[ | | |  |
| 0.0075 | CRI | 0.817 | 0.827 | 0.832 | 0.822 | 0.810 | 0.814 |  |  |  |  |  |  |  |  |  |  |  | [0.860-0.889] | | |  |
| 0.0325 | CUB | 0.800 | 0.811 | 0.818 | 0.807 | 0.791 | 0.799 | 0.775 |  |  |  |  |  |  |  |  |  |  |  |  |  |  |
| 0.0002 | DMA | 0.803 | 0.822 | 0.828 | 0.826 | 0.803 | 0.815 | 0.796 | 0.791 |  |  |  |  |  |  |  |  |  |  |  |  |  |
| 0.0143 | DOM | 0.826 | 0.837 | 0.839 | 0.843 | 0.818 | 0.833 | 0.817 | 0.819 | 0.827 |  |  |  |  |  |  |  |  |  |  |  |  |
| 0.0222 | ECU | 0.833 | 0.839 | 0.843 | 0.841 | 0.825 | 0.835 | 0.821 | 0.830 | 0.839 | 0.843 |  |  |  |  |  |  |  |  |  |  |  |
| 0.0128 | SLV | 0.863 | 0.863 | 0.864 | 0.872 | 0.851 | 0.865 | 0.853 | 0.857 | 0.853 | 0.861 | 0.853 |  |  |  |  |  |  |  |  |  |  |
| 0.0003 | GRD | 0.808 | 0.832 | 0.834 | 0.830 | 0.812 | 0.820 | 0.805 | 0.808 | 0.828 | 0.835 | 0.863 | 0.813 |  |  |  |  |  |  |  |  |  |
| 0.0016 | GUY | 0.809 | 0.831 | 0.830 | 0.834 | 0.804 | 0.821 | 0.803 | 0.804 | 0.816 | 0.830 | 0.848 | 0.812 | 0.797 |  |  |  |  |  |  |  |  |
| 0.2230 | MEX | 0.830 | 0.849 | 0.850 | 0.850 | 0.831 | 0.840 | 0.831 | 0.831 | 0.843 | 0.848 | 0.869 | 0.833 | 0.826 | 0.840 |  |  |  |  |  |  |  |
| 0.0074 | NIC | 0.824 | 0.842 | 0.844 | 0.844 | 0.822 | 0.832 | 0.821 | 0.820 | 0.835 | 0.842 | 0.857 | 0.826 | 0.818 | 0.839 | 0.828 |  |  |  |  |  |  |
| 0.0064 | PAN | 0.815 | 0.830 | 0.834 | 0.832 | 0.811 | 0.822 | 0.805 | 0.809 | 0.826 | 0.835 | 0.856 | 0.818 | 0.812 | 0.837 | 0.827 | 0.818 |  |  |  |  |  |
| 0.0099 | PRY | 0.830 | 0.839 | 0.843 | 0.843 | 0.825 | 0.836 | 0.821 | 0.827 | 0.838 | 0.843 | 0.860 | 0.833 | 0.828 | 0.847 | 0.841 | 0.833 | 0.842 |  |  |  |  |
| 0.0374 | PER | 0.863 | 0.849 | 0.860 | 0.853 | 0.847 | 0.851 | 0.843 | 0.858 | 0.866 | 0.863 | 0.889 | 0.861 | 0.862 | 0.868 | 0.869 | 0.861 | 0.865 | 0.855 |  |  |  |
| 0.0004 | LCA | 0.813 | 0.828 | 0.833 | 0.825 | 0.811 | 0.817 | 0.802 | 0.812 | 0.830 | 0.834 | 0.864 | 0.817 | 0.817 | 0.837 | 0.831 | 0.820 | 0.834 | 0.854 | 0.815 |  |  |
| 0.0003 | VCT | 0.806 | 0.825 | 0.829 | 0.820 | 0.807 | 0.812 | 0.795 | 0.805 | 0.827 | 0.831 | 0.862 | 0.812 | 0.814 | 0.836 | 0.828 | 0.815 | 0.830 | 0.856 | 0.811 | 0.803 |  |
| 0.0097 | URY | 0.838 | 0.841 | 0.847 | 0.837 | 0.832 | 0.834 | 0.818 | 0.840 | 0.851 | 0.849 | 0.872 | 0.843 | 0.846 | 0.863 | 0.856 | 0.842 | 0.850 | 0.864 | 0.836 | 0.830 | 0.840 |

Source: WHO Mortality Database [20] and World Population Prospects [21]. Note: ATG - Antigua and Barbuda, ARG - Argentina, BRA - Brazil, CHL - Chile, COL - Colombia, CRI - Costa Rica, CUB - Cuba, DMA - Dominica, DOM - Dominican Republic, ECU - Ecuador, SLV - El Salvador, GRD - Grenada, GUY - Guyana, MEX - Mexico, NIC - Nicaragua, PAN - Panama, PRY - Paraguay, PER - Peru, LCA - Saint Lucia, VCT - Saint Vincent and Grenadines, URY - Uruguay.

# **Figure 6A – Correlation between the CoD diversity within a country and its contribution to overall CoD diversity in the LAC region (2000 to 2018)**


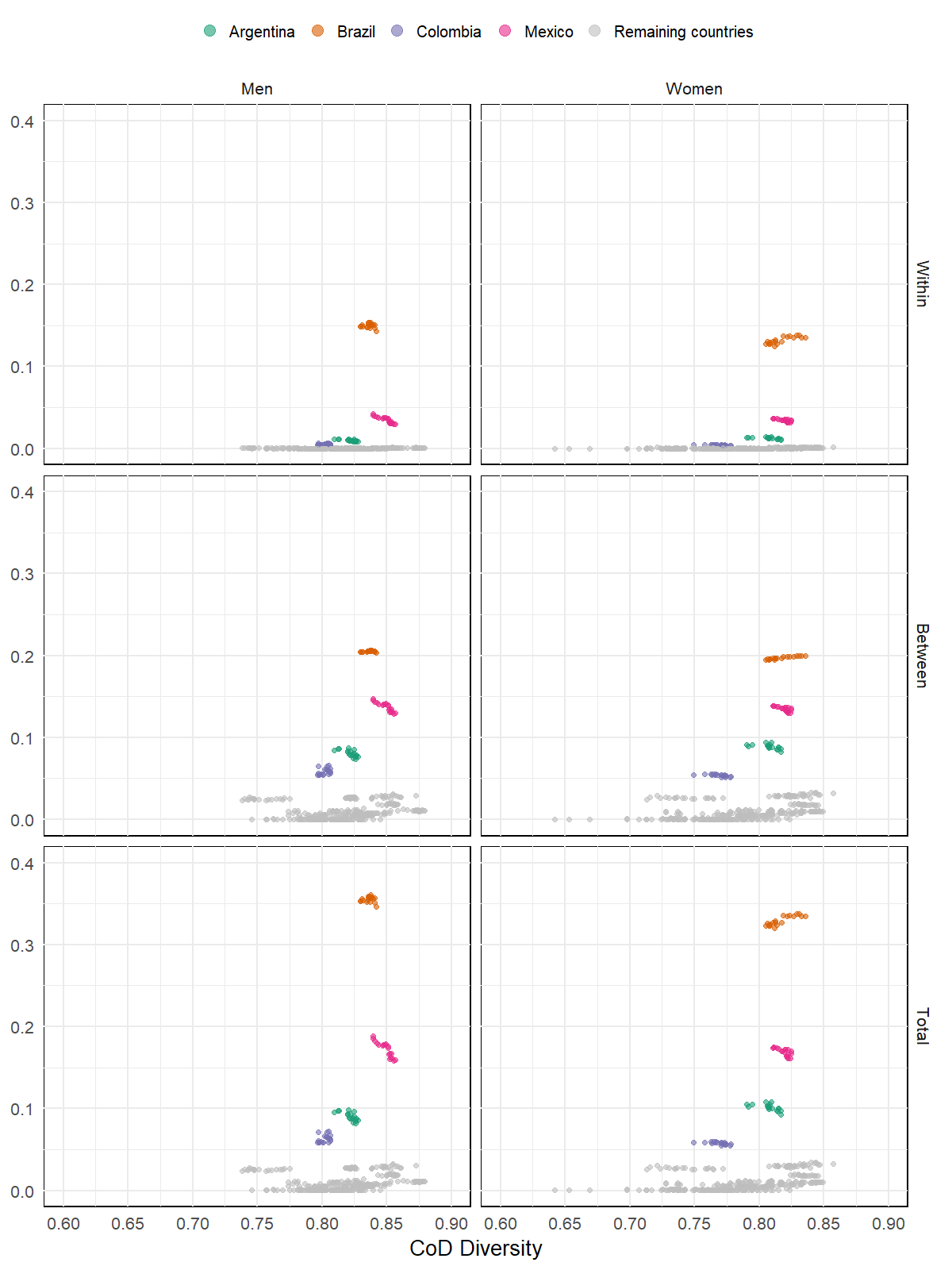


Source: WHO Mortality Database [20] and World Population Prospects [21].

# **Figure 7A – Correlation between the proportion of CoD within a country and its contribution to overall CoD diversity in the LAC region (2000 to 2018)**

#
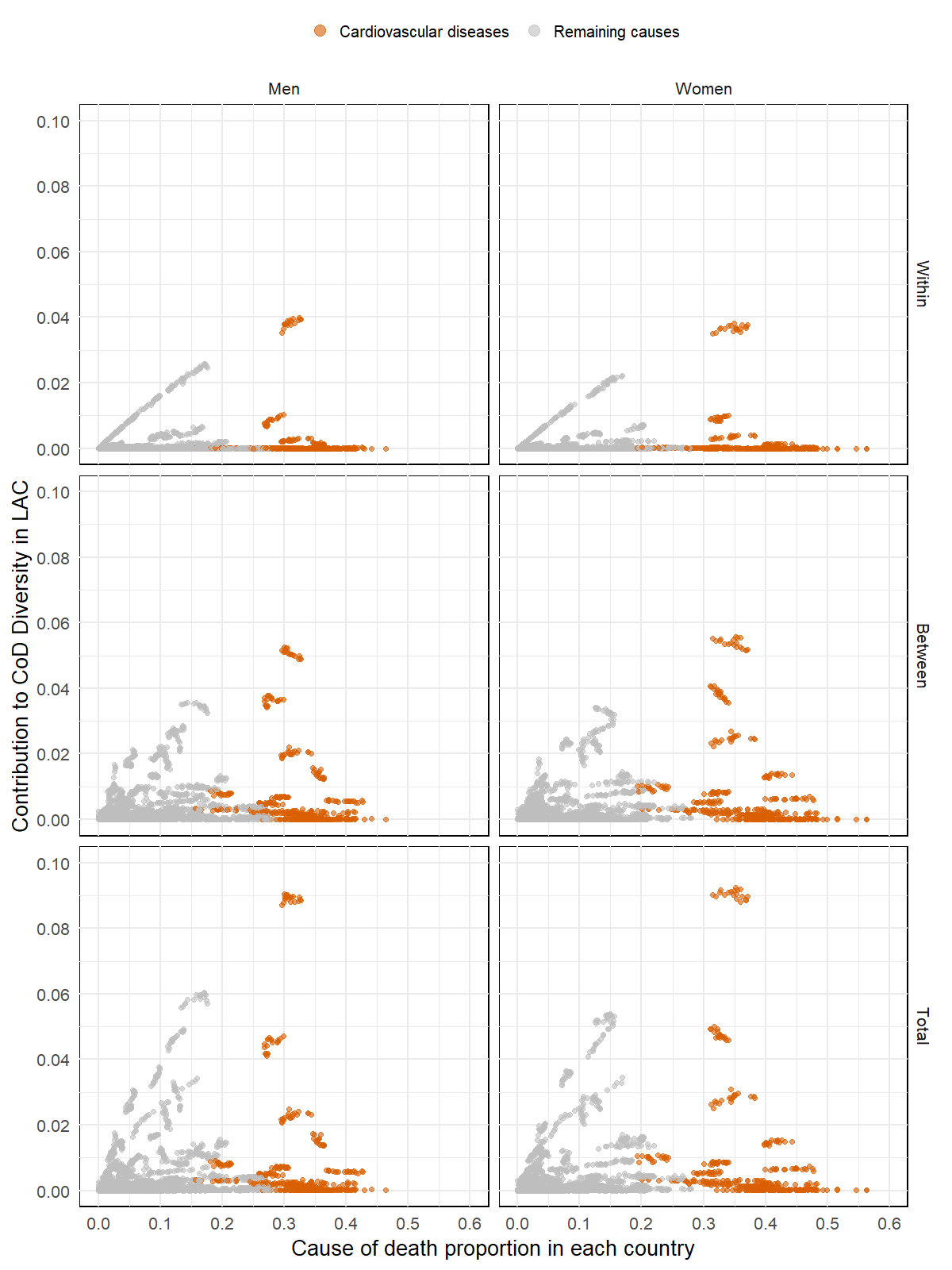


Source: WHO Mortality Database [20] and World Population Prospects [21].

**Table 1A - Countries' contributions to CoD diversity in the LAC region and its components (2000 and 2018)**

| **Year** | **Sex** | **Country** | **Between** | **Within** | **Total contribution** |
| --- | --- | --- | --- | --- | --- |
| 2000 | Men | Antigua and Barbuda | 0.000(0.000-0.000) | 0.000(0.000-0.000) | 0.000(0.000-0.000) |
| 2000 | Men | Argentina | 0.085(0.083-0.086) | 0.011(0.011-0.011) | 0.095(0.094-0.097) |
| 2000 | Men | Brazil | 0.206(0.202-0.209) | 0.148(0.146-0.150) | 0.354(0.348-0.360) |
| 2000 | Men | Chile | 0.026(0.026-0.027) | 0.001(0.001-0.001) | 0.027(0.027-0.028) |
| 2000 | Men | Colombia | 0.065(0.064-0.066) | 0.006(0.006-0.006) | 0.071(0.070-0.073) |
| 2000 | Men | Costa Rica | 0.005(0.005-0.005) | 0.000(0.000-0.000) | 0.005(0.005-0.005) |
| 2000 | Men | Cuba | 0.027(0.026-0.027) | 0.001(0.001-0.001) | 0.028(0.027-0.028) |
| 2000 | Men | Dominica | 0.000(0.000-0.000) | 0.000(0.000-0.000) | 0.000(0.000-0.000) |
| 2000 | Men | Dominican Republic | 0.009(0.009-0.009) | 0.000(0.000-0.000) | 0.009(0.009-0.009) |
| 2000 | Men | Ecuador | 0.020(0.020-0.020) | 0.001(0.000-0.001) | 0.021(0.020-0.021) |
| 2000 | Men | El Salvador | 0.011(0.011-0.011) | 0.000(0.000-0.000) | 0.011(0.011-0.011) |
| 2000 | Men | Grenada | 0.000(0.000-0.000) | 0.000(0.000-0.000) | 0.000(0.000-0.000) |
| 2000 | Men | Guyana | 0.002(0.002-0.002) | 0.000(0.000-0.000) | 0.002(0.002-0.002) |
| 2000 | Men | Mexico | 0.130(0.128-0.132) | 0.030(0.029-0.030) | 0.159(0.157-0.162) |
| 2000 | Men | Nicaragua | 0.005(0.005-0.005) | 0.000(0.000-0.000) | 0.005(0.005-0.005) |
| 2000 | Men | Panama | 0.004(0.004-0.004) | 0.000(0.000-0.000) | 0.004(0.004-0.004) |
| 2000 | Men | Paraguay | 0.007(0.007-0.007) | 0.000(0.000-0.000) | 0.007(0.007-0.007) |
| 2000 | Men | Peru | 0.029(0.029-0.030) | 0.001(0.001-0.001) | 0.030(0.030-0.031) |
| 2000 | Men | Saint Lucia | 0.000(0.000-0.000) | 0.000(0.000-0.000) | 0.000(0.000-0.000) |
| 2000 | Men | St. Vincent and the Grenadines | 0.000(0.000-0.000) | 0.000(0.000-0.000) | 0.000(0.000-0.000) |
| 2000 | Men | Uruguay | 0.010(0.010-0.010) | 0.000(0.000-0.000) | 0.010(0.010-0.011) |
| 2000 | Women | Antigua and Barbuda | 0.000(0.000-0.000) | 0.000(0.000-0.000) | 0.000(0.000-0.000) |
| 2000 | Women | Argentina | 0.090(0.088-0.091) | 0.013(0.013-0.013) | 0.102(0.100-0.104) |
| 2000 | Women | Brazil | 0.195(0.191-0.199) | 0.128(0.125-0.130) | 0.323(0.317-0.329) |
| 2000 | Women | Chile | 0.028(0.028-0.029) | 0.001(0.001-0.001) | 0.029(0.029-0.030) |
| 2000 | Women | Colombia | 0.055(0.054-0.056) | 0.004(0.004-0.004) | 0.059(0.058-0.060) |
| 2000 | Women | Costa Rica | 0.005(0.005-0.005) | 0.000(0.000-0.000) | 0.005(0.005-0.005) |
| 2000 | Women | Cuba | 0.029(0.029-0.030) | 0.001(0.001-0.001) | 0.030(0.030-0.031) |
| 2000 | Women | Dominica | 0.000(0.000-0.000) | 0.000(0.000-0.000) | 0.000(0.000-0.000) |
| 2000 | Women | Dominican Republic | 0.008(0.008-0.008) | 0.000(0.000-0.000) | 0.008(0.008-0.008) |
| 2000 | Women | Ecuador | 0.020(0.019-0.020) | 0.001(0.000-0.001) | 0.020(0.020-0.021) |
| 2000 | Women | El Salvador | 0.010(0.009-0.010) | 0.000(0.000-0.000) | 0.010(0.009-0.010) |
| 2000 | Women | Grenada | 0.000(0.000-0.000) | 0.000(0.000-0.000) | 0.000(0.000-0.000) |
| 2000 | Women | Guyana | 0.002(0.002-0.002) | 0.000(0.000-0.000) | 0.002(0.002-0.002) |
| 2000 | Women | Mexico | 0.130(0.127-0.132) | 0.031(0.031-0.032) | 0.161(0.158-0.164) |
| 2000 | Women | Nicaragua | 0.005(0.005-0.005) | 0.000(0.000-0.000) | 0.005(0.005-0.005) |
| 2000 | Women | Panama | 0.004(0.004-0.004) | 0.000(0.000-0.000) | 0.004(0.004-0.004) |
| 2000 | Women | Paraguay | 0.007(0.007-0.007) | 0.000(0.000-0.000) | 0.007(0.007-0.007) |
| 2000 | Women | Peru | 0.032(0.031-0.032) | 0.001(0.001-0.001) | 0.033(0.032-0.033) |
| 2000 | Women | Saint Lucia | 0.000(0.000-0.000) | 0.000(0.000-0.000) | 0.000(0.000-0.000) |
| 2000 | Women | St. Vincent and the Grenadines | 0.000(0.000-0.000) | 0.000(0.000-0.000) | 0.000(0.000-0.000) |
| 2000 | Women | Uruguay | 0.011(0.011-0.011) | 0.000(0.000-0.000) | 0.011(0.011-0.012) |
| 2018 | Men | Antigua and Barbuda | 0.000(0.000-0.000) | 0.000(0.000-0.000) | 0.000(0.000-0.000) |
| 2018 | Men | Argentina | 0.074(0.073-0.075) | 0.008(0.008-0.008) | 0.082(0.081-0.083) |
| 2018 | Men | Brazil | 0.204(0.201-0.207) | 0.143(0.141-0.145) | 0.346(0.341-0.351) |
| 2018 | Men | Chile | 0.026(0.025-0.026) | 0.001(0.001-0.001) | 0.026(0.026-0.027) |
| 2018 | Men | Colombia | 0.056(0.055-0.057) | 0.004(0.004-0.004) | 0.060(0.060-0.061) |
| 2018 | Men | Costa Rica | 0.006(0.006-0.006) | 0.000(0.000-0.000) | 0.006(0.006-0.006) |
| 2018 | Men | Cuba | 0.026(0.025-0.026) | 0.001(0.001-0.001) | 0.027(0.026-0.027) |
| 2018 | Men | Dominica | 0.000(0.000-0.000) | 0.000(0.000-0.000) | 0.000(0.000-0.000) |
| 2018 | Men | Dominican Republic | 0.012(0.012-0.012) | 0.000(0.000-0.000) | 0.012(0.012-0.012) |
| 2018 | Men | Ecuador | 0.018(0.018-0.019) | 0.000(0.000-0.000) | 0.019(0.019-0.019) |
| 2018 | Men | El Salvador | 0.011(0.011-0.011) | 0.000(0.000-0.000) | 0.011(0.011-0.011) |
| 2018 | Men | Grenada | 0.000(0.000-0.000) | 0.000(0.000-0.000) | 0.000(0.000-0.000) |
| 2018 | Men | Guyana | 0.001(0.001-0.001) | 0.000(0.000-0.000) | 0.001(0.001-0.001) |
| 2018 | Men | Mexico | 0.147(0.145-0.149) | 0.042(0.041-0.042) | 0.189(0.186-0.191) |
| 2018 | Men | Nicaragua | 0.006(0.006-0.006) | 0.000(0.000-0.000) | 0.006(0.006-0.006) |
| 2018 | Men | Panama | 0.005(0.005-0.005) | 0.000(0.000-0.000) | 0.005(0.005-0.005) |
| 2018 | Men | Paraguay | 0.008(0.008-0.008) | 0.000(0.000-0.000) | 0.008(0.008-0.008) |
| 2018 | Men | Peru | 0.031(0.031-0.031) | 0.001(0.001-0.001) | 0.032(0.032-0.033) |
| 2018 | Men | Saint Lucia | 0.000(0.000-0.000) | 0.000(0.000-0.000) | 0.000(0.000-0.000) |
| 2018 | Men | St. Vincent and the Grenadines | 0.000(0.000-0.000) | 0.000(0.000-0.000) | 0.000(0.000-0.000) |
| 2018 | Men | Uruguay | 0.008(0.008-0.008) | 0.000(0.000-0.000) | 0.008(0.008-0.008) |
| 2018 | Women | Antigua and Barbuda | 0.000(0.000-0.000) | 0.000(0.000-0.000) | 0.000(0.000-0.000) |
| 2018 | Women | Argentina | 0.083(0.081-0.084) | 0.010(0.010-0.010) | 0.093(0.091-0.094) |
| 2018 | Women | Brazil | 0.200(0.196-0.203) | 0.135(0.133-0.138) | 0.335(0.330-0.340) |
| 2018 | Women | Chile | 0.028(0.028-0.029) | 0.001(0.001-0.001) | 0.029(0.029-0.030) |
| 2018 | Women | Colombia | 0.055(0.054-0.056) | 0.004(0.004-0.004) | 0.059(0.058-0.060) |
| 2018 | Women | Costa Rica | 0.006(0.006-0.006) | 0.000(0.000-0.000) | 0.006(0.006-0.006) |
| 2018 | Women | Cuba | 0.026(0.026-0.027) | 0.001(0.001-0.001) | 0.027(0.027-0.027) |
| 2018 | Women | Dominica | 0.000(0.000-0.000) | 0.000(0.000-0.000) | 0.000(0.000-0.000) |
| 2018 | Women | Dominican Republic | 0.010(0.010-0.010) | 0.000(0.000-0.000) | 0.010(0.010-0.010) |
| 2018 | Women | Ecuador | 0.018(0.018-0.018) | 0.000(0.000-0.000) | 0.019(0.018-0.019) |
| 2018 | Women | El Salvador | 0.010(0.010-0.010) | 0.000(0.000-0.000) | 0.010(0.010-0.010) |
| 2018 | Women | Grenada | 0.000(0.000-0.000) | 0.000(0.000-0.000) | 0.000(0.000-0.000) |
| 2018 | Women | Guyana | 0.001(0.001-0.001) | 0.000(0.000-0.000) | 0.001(0.001-0.001) |
| 2018 | Women | Mexico | 0.139(0.137-0.141) | 0.037(0.036-0.037) | 0.175(0.173-0.178) |
| 2018 | Women | Nicaragua | 0.006(0.006-0.006) | 0.000(0.000-0.000) | 0.006(0.006-0.006) |
| 2018 | Women | Panama | 0.005(0.005-0.005) | 0.000(0.000-0.000) | 0.005(0.005-0.005) |
| 2018 | Women | Paraguay | 0.008(0.008-0.008) | 0.000(0.000-0.000) | 0.008(0.008-0.008) |
| 2018 | Women | Peru | 0.033(0.033-0.034) | 0.001(0.001-0.001) | 0.035(0.034-0.035) |
| 2018 | Women | Saint Lucia | 0.000(0.000-0.000) | 0.000(0.000-0.000) | 0.000(0.000-0.000) |
| 2018 | Women | St. Vincent and the Grenadines | 0.000(0.000-0.000) | 0.000(0.000-0.000) | 0.000(0.000-0.000) |
| 2018 | Women | Uruguay | 0.010(0.010-0.010) | 0.000(0.000-0.000) | 0.010(0.010-0.010) |

Source: WHO Mortality Database [20] and World Population Prospects [21]. Note: Confidence interval at a 95% significance level in parenthesis.

**Table 2A - Causes of death contribution to CoD diversity in the LAC region and its components (2000 and 2018)**

| **Year** | **Sex** | **ICD-10 chapters** | **Between** | **Within** | **Total contribution** |
| --- | --- | --- | --- | --- | --- |
| 2000 | Men | Infectious diseases | 0.027(0.026-0.028) | 0.008(0.008-0.009) | 0.035(0.035-0.036) |
| 2000 | Men | Neoplasms | 0.101(0.100-0.102) | 0.028(0.028-0.029) | 0.129(0.128-0.131) |
| 2000 | Men | Blood diseases | 0.004(0.004-0.004) | 0.001(0.001-0.001) | 0.005(0.005-0.005) |
| 2000 | Men | Endocrine diseases | 0.043(0.042-0.044) | 0.012(0.012-0.013) | 0.056(0.055-0.057) |
| 2000 | Men | Mental disorders | 0.007(0.007-0.008) | 0.002(0.002-0.002) | 0.009(0.009-0.010) |
| 2000 | Men | Neurological diseases | 0.009(0.009-0.010) | 0.003(0.002-0.003) | 0.012(0.011-0.012) |
| 2000 | Men | Diseases of the eye and ear | 0.000(0.000-0.000) | 0.000(0.000-0.000) | 0.000(0.000-0.000) |
| 2000 | Men | Cardiovascular diseases | 0.163(0.162-0.164) | 0.050(0.050-0.050) | 0.213(0.212-0.214) |
| 2000 | Men | Respiratory diseases | 0.078(0.077-0.079) | 0.024(0.024-0.024) | 0.101(0.100-0.103) |
| 2000 | Men | Digestive diseases | 0.045(0.044-0.045) | 0.012(0.012-0.013) | 0.057(0.056-0.058) |
| 2000 | Men | Skin diseases | 0.002(0.001-0.002) | 0.000(0.000-0.000) | 0.002(0.002-0.002) |
| 2000 | Men | Musculoskeletal diseases | 0.002(0.002-0.002) | 0.000(0.000-0.001) | 0.002(0.002-0.003) |
| 2000 | Men | Genitourinary diseases | 0.018(0.018-0.019) | 0.005(0.004-0.005) | 0.023(0.022-0.024) |
| 2000 | Men | Pregnancy, childbirth and the puerperium | 0.000(0.000-0.000) | 0.000(0.000-0.000) | 0.000(0.000-0.000) |
| 2000 | Men | Perinatal conditions | 0.010(0.010-0.011) | 0.004(0.004-0.004) | 0.014(0.013-0.015) |
| 2000 | Men | Congenital malformations | 0.004(0.004-0.004) | 0.001(0.001-0.001) | 0.005(0.005-0.005) |
| 2000 | Men | Ill-defined causes | 0.062(0.061-0.062) | 0.026(0.026-0.026) | 0.087(0.086-0.089) |
| 2000 | Men | External causes | 0.068(0.067-0.069) | 0.021(0.020-0.021) | 0.089(0.088-0.090) |
| 2000 | Women | Infectious diseases | 0.024(0.023-0.025) | 0.007(0.007-0.007) | 0.031(0.030-0.032) |
| 2000 | Women | Neoplasms | 0.100(0.099-0.101) | 0.026(0.025-0.026) | 0.126(0.124-0.127) |
| 2000 | Women | Blood diseases | 0.005(0.005-0.005) | 0.001(0.001-0.001) | 0.006(0.006-0.007) |
| 2000 | Women | Endocrine diseases | 0.062(0.061-0.063) | 0.017(0.017-0.018) | 0.079(0.078-0.081) |
| 2000 | Women | Mental disorders | 0.005(0.005-0.006) | 0.001(0.001-0.001) | 0.006(0.006-0.006) |
| 2000 | Women | Neurological diseases | 0.010(0.009-0.010) | 0.003(0.002-0.003) | 0.012(0.012-0.013) |
| 2000 | Women | Diseases of the eye and ear | 0.000 (0.000-0.000) | 0.000(0.000-0.000) | 0.000(0.000-0.000) |
| 2000 | Women | Cardiovascular diseases | 0.179(0.178-0.180) | 0.051(0.051-0.052) | 0.230(0.229-0.232) |
| 2000 | Women | Respiratory diseases | 0.080(0.079-0.081) | 0.023(0.022-0.023) | 0.102(0.101-0.104) |
| 2000 | Women | Digestive diseases | 0.035(0.034-0.036) | 0.009(0.009-0.010) | 0.045(0.043-0.046) |
| 2000 | Women | Skin diseases | 0.003(0.003-0.003) | 0.001(0.001-0.001) | 0.004(0.003-0.004) |
| 2000 | Women | Musculoskeletal diseases | 0.004(0.004-0.005) | 0.001(0.001-0.001) | 0.005(0.005-0.006) |
| 2000 | Women | Genitourinary diseases | 0.018(0.017-0.019) | 0.004(0.004-0.005) | 0.022(0.022-0.023) |
| 2000 | Women | Pregnancy, childbirth and the puerperium | 0.001(0.001-0.002) | 0.000(0.000-0.000) | 0.002(0.001-0.002) |
| 2000 | Women | Perinatal conditions | 0.008(0.007-0.008) | 0.003(0.003-0.003) | 0.011(0.010-0.011) |
| 2000 | Women | Congenital malformations | 0.004(0.003-0.004) | 0.001(0.001-0.001) | 0.005(0.004-0.005) |
| 2000 | Women | Ill-defined causes | 0.068(0.067-0.069) | 0.025(0.025-0.025) | 0.093(0.092-0.095) |
| 2000 | Women | External causes | 0.025(0.024-0.025) | 0.007(0.006-0.007) | 0.031(0.030-0.032) |
| 2018 | Men | Infectious diseases | 0.023(0.022-0.023) | 0.008(0.008-0.008) | 0.031(0.030-0.031) |
| 2018 | Men | Neoplasms | 0.107(0.106-0.108) | 0.033(0.033-0.033) | 0.140(0.139-0.141) |
| 2018 | Men | Blood diseases | 0.003 (0.003-0.004) | 0.001(0.001-0.001) | 0.005(0.004-0.005) |
| 2018 | Men | Endocrine diseases | 0.053(0.052-0.053) | 0.016(0.016-0.017) | 0.069(0.068-0.070) |
| 2018 | Men | Mental disorders | 0.009(0.008-0.009) | 0.002(0.002-0.002) | 0.011(0.010-0.011) |
| 2018 | Men | Neurological diseases | 0.016(0.016-0.017) | 0.006(0.006-0.006) | 0.022(0.022-0.023) |
| 2018 | Men | Diseases of the eye and ear | 0.000(0.000-0.000) | 0.000(0.000-0.000) | 0.000(0.000-0.000) |
| 2018 | Men | Cardiovascular diseases | 0.160(0.160-0.161) | 0.050(0.050-0.050) | 0.210(0.209-0.211) |
| 2018 | Men | Respiratory diseases | 0.090(0.089-0.091) | 0.027(0.027-0.028) | 0.118(0.116-0.119) |
| 2018 | Men | Digestive diseases | 0.046(0.046-0.047) | 0.014(0.014-0.014) | 0.060(0.059-0.061) |
| 2018 | Men | Skin diseases | 0.004(0.003-0.004) | 0.001(0.001-0.001) | 0.005(0.004-0.005) |
| 2018 | Men | Musculoskeletal diseases | 0.003(0.003-0.003) | 0.001(0.001-0.001) | 0.004(0.003-0.004) |
| 2018 | Men | Genitourinary diseases | 0.028(0.028-0.029) | 0.008(0.008-0.009) | 0.037(0.036-0.037) |
| 2018 | Men | Pregnancy, childbirth and the puerperium | 0.000(0.000-0.000) | 0.000(0.000-0.000) | 0.000(0.000-0.000) |
| 2018 | Men | Perinatal conditions | 0.006(0.005-0.006) | 0.002(0.002-0.002) | 0.008(0.007-0.008) |
| 2018 | Men | Congenital malformations | 0.003(0.003-0.004) | 0.001(0.001-0.001) | 0.004(0.004-0.005) |
| 2018 | Men | Ill-defined causes | 0.030(0.029-0.031) | 0.010(0.010-0.011) | 0.040(0.040-0.041) |
| 2018 | Men | External causes | 0.059(0.058-0.059) | 0.019(0.018-0.019) | 0.077(0.076-0.078) |
| 2018 | Women | Infectious diseases | 0.021(0.021-0.022) | 0.007(0.007-0.008) | 0.029(0.028-0.030) |
| 2018 | Women | Neoplasms | 0.103(0.102-0.104) | 0.030(0.029-0.030) | 0.132(0.131-0.133) |
| 2018 | Women | Blood diseases | 0.004(0.004-0.005) | 0.001(0.001-0.001) | 0.006(0.005-0.006) |
| 2018 | Women | Endocrine diseases | 0.065(0.064-0.066) | 0.019(0.019-0.020) | 0.084(0.083-0.085) |
| 2018 | Women | Mental disorders | 0.009(0.009-0.010) | 0.002(0.002-0.002) | 0.011(0.010-0.011) |
| 2018 | Women | Neurological diseases | 0.021(0.021-0.022) | 0.008(0.008-0.009) | 0.030(0.029-0.030) |
| 2018 | Women | Diseases of the eye and ear | 0.000(0.000-0.000) | 0.000(0.000-0.000) | 0.000(0.000-0.000) |
| 2018 | Women | Cardiovascular diseases | 0.170(0.169-0.171) | 0.050(0.050-0.050) | 0.220(0.219-0.221) |
| 2018 | Women | Respiratory diseases | 0.099(0.098-0.100) | 0.029(0.028-0.029) | 0.127(0.126-0.129) |
| 2018 | Women | Digestive diseases | 0.039(0.038-0.040) | 0.011(0.011-0.011) | 0.050(0.049-0.051) |
| 2018 | Women | Skin diseases | 0.006(0.005-0.006) | 0.002(0.002-0.002) | 0.007(0.007-0.008) |
| 2018 | Women | Musculoskeletal diseases | 0.006(0.006-0.006) | 0.002(0.001-0.002) | 0.008(0.007-0.008) |
| 2018 | Women | Genitourinary diseases | 0.029(0.029-0.030) | 0.009(0.009-0.010) | 0.039(0.038-0.040) |
| 2018 | Women | Pregnancy, childbirth and the puerperium | 0.001(0.001-0.001) | 0.000(0.000-0.000) | 0.001(0.001-0.001) |
| 2018 | Women | Perinatal conditions | 0.004(0.004-0.005) | 0.001(0.001-0.002) | 0.006(0.005-0.006) |
| 2018 | Women | Congenital malformations | 0.003(0.003-0.003) | 0.001(0.001-0.001) | 0.004(0.004-0.004) |
| 2018 | Women | Ill-defined causes | 0.032(0.031-0.033) | 0.010(0.010-0.010) | 0.042(0.041-0.043) |
| 2018 | Women | External causes | 0.025(0.024-0.025) | 0.008(0.007-0.008) | 0.032(0.031-0.033) |

Source: WHO Mortality Database [20] and World Population Prospects [21]. Note: Confidence interval at a 95% significance level in parenthesis.
